# Behavioural and neural indices of perceptual decision-making in autistic children during visual motion tasks

**DOI:** 10.1038/s41598-022-09885-4

**Published:** 2022-04-12

**Authors:** Catherine Manning, Cameron D. Hassall, Laurence T. Hunt, Anthony M. Norcia, Eric-Jan Wagenmakers, Nathan J. Evans, Gaia Scerif

**Affiliations:** 1 Department of Experimental Psychology, University of Oxford, Oxford, UK; 2 School of Psychology and Clinical Language Sciences, University of Reading, Reading, UK; 3 Department of Psychiatry, University of Oxford, Oxford, UK; 4 Department of Psychology, Stanford University, Stanford, USA; 5 Faculty of Social and Behavioural Sciences, University of Amsterdam, Amsterdam, The Netherlands; 6 School of Psychology, University of Queensland, Brisbane, Australia

**Keywords:** Decision, Perception, Cognitive neuroscience, Computational neuroscience, Sensory processing, Visual system, Motion detection, Neuroscience, Psychology, Human behaviour

## Abstract

Many studies report atypical responses to sensory information in autistic individuals, yet it is not clear which stages of processing are affected, with little consideration given to decision-making processes. We combined diffusion modelling with high-density EEG to identify which processing stages differ between 50 autistic and 50 typically developing children aged 6–14 years during two visual motion tasks. Our pre-registered hypotheses were that autistic children would show task-dependent differences in sensory evidence accumulation, alongside a more cautious decision-making style and longer non-decision time across tasks. We tested these hypotheses using hierarchical Bayesian diffusion models with a rigorous blind modelling approach, finding no conclusive evidence for our hypotheses. Using a data-driven method, we identified a response-locked centro-parietal component previously linked to the decision-making process. The build-up in this component did not consistently relate to evidence accumulation in autistic children. This suggests that the relationship between the EEG measure and diffusion-modelling is not straightforward in autistic children. Compared to a related study of children with dyslexia, motion processing differences appear less pronounced in autistic children. Exploratory analyses also suggest weak evidence that ADHD symptoms moderate perceptual decision-making in autistic children.

## Introduction

Autism is a developmental condition affecting social communication and interaction as well as non-social domains, such as sensory functioning^1^. Atypical responses to sensory information are well-documented in autism, ranging from first-hand reports of sensory processing differences, e.g.,^2–4^ to evidence of altered behavioural and physiological responses to sensory stimuli (see Ref.^5^, for review). Yet, the reasons for atypical sensory functioning in autism remain unclear. An extensive literature has aimed to better understand sensory processing in autism by measuring psychophysical thresholds. Here, researchers estimate the stimulus level (or difference between stimuli) required to give rise to a given level of accuracy for each individual, and compare these estimates between autistic and non-autistic individuals. Reports of group differences in psychophysical thresholds range from reduced sensitivity, e.g.,^6–8^ to enhanced sensitivity, e.g.,^9–11^, depending on the task. While there is currently a lack of evidence linking differences in psychophysical thresholds to the everyday sensory symptoms experienced by autistic individuals^12^, differences in sensitivity nonetheless show that autistic people process sensory information differently to those without autism.

Visual motion processing is one aspect of sensory processing that has been extensively studied in autism^13,14^. Visual motion processing is an important function enabling individuals to interact with dynamic objects, segment scenes and perceive depth^15^; therefore differences in visual motion processing are likely to have considerable impacts on everyday life. It has been suggested that motion processing might be impaired in developmental conditions such as autism, because these tasks rely heavily on the dorsal stream—a pathway in the brain which has been proposed to be particularly vulnerable to atypical development^15^. Accordingly, reduced sensitivity in autistic individuals compared to non-autistic individuals has been reported in a range of visual motion tasks^14,16,17^. However, in other tasks, sensitivity has even been shown to be *increased* in autistic compared to non-autistic participants^18,19^, which is difficult to reconcile with the dorsal-stream vulnerability account.

One factor that might explain discrepant results across tasks is the extent to which the tasks tax integration and segregation processes^20–22^. Autistic individuals have been shown to be less sensitive than non-autistic individuals in the motion coherence task (see Ref.^14^, for review), which requires participants to discriminate the direction of motion carried by coherently moving signal dots in a field of randomly moving noise dots^16–18^. This task not only involves integrating over dots to see the overall motion, but also requires segregating signal dots from noise dots. Yet, autistic children have been shown to perform *better* than typically developing children in a direction integration task which does not require segregating signal dots from noise dots^18,23^. In this task, participants were required to discriminate the average direction of dots moving in directions sampled from a Gaussian distribution, with difficulty being manipulated by increasing the variability of dot directions. Therefore, it seems that autistic children demonstrate increased integration of motion information compared to typically developing children, but may be limited in the motion coherence task due to difficulties filtering out the randomly moving noise dots (‘noise exclusion’; see also Refs.^24,25^). Difficulties in filtering task-irrelevant information could be related to feelings of sensory overload in autistic individuals^18^.

Yet, so far, studies of motion processing have failed to fully consider the dynamic processes leading up to responses, so that it is not clear at which level of processing group differences in sensitivity arise. Resolving this will help us better understand the mechanisms underlying atypical sensory processing in autism. In non-human primates, neurons in the middle temporal area (MT/V5) are specialised for processing motion signals^26^, and these motion signals are then integrated in parietal areas as part of the decision-making process, until enough evidence has been accumulated for the animal to reach a decision^27,28^. Similarly, electroencephalography (EEG) studies in humans have shown a build-up of activity over centro-parietal electrodes that has been linked to the decision-making process during motion tasks, in both adults^29,30^ and children^31,32^. A recent study comparing evoked potentials locked to the onset of directional motion in autistic and typically developing children suggested no differences in early neural responses to directional motion between autistic and typically developing children, but suggested that later processing stages may be affected^22^. Therefore, it might be that early sensory processing of motion information is unaffected in autism, but that there are instead differences in decision-making processes.

**Figure 1:**
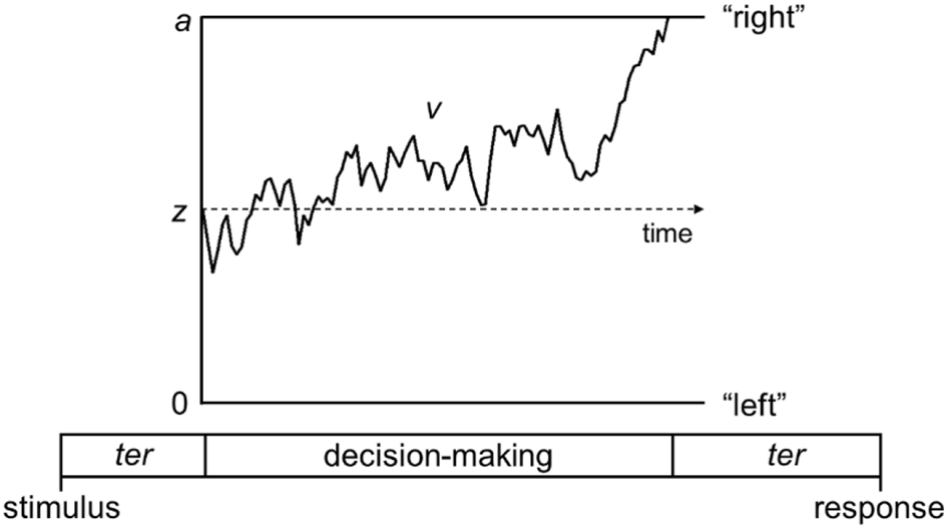
Schematic representation of the main diffusion model parameters. Within the diffusion model, the decision-making process is represented as a noisy evidence accumulation process originating from a starting point, *z*, towards one of two decision bounds (e.g., “left” and “right” directions in the motion tasks used in this study). Boundary separation, *a,* reflects the distance between the two bounds, indexing response caution. Drift-rate, *v,* is the rate of evidence accumulation. Non-decision time, *ter*, is the time taken for non-decision processes including sensory encoding and response generation. Figure reproduced from https://osf.io/rqw62/ under a CC-BY4.0 License.

One way to study decision-making processes more carefully is to use diffusion models, which analyse both accuracy and response time in order to decompose performance into distinct processing stages^33,34^ (see Ref.^35^, for review and Fig. 1 for a graphical representation of multiple parameters feeding into decisions). The decision is modelled as a noisy evidence accumulation process from a starting point towards one of two decision bounds (e.g., “left” or “right” decision bounds for a motion discrimination task). The main parameters of the model are drift-rate, boundary separation, starting point and non-decision time. The drift-rate reflects the rate of evidence accumulation, with information being accumulated more rapidly in more sensitive individuals and in conditions with stronger sensory evidence. Boundary separation reflects how far apart the decision boundaries are, and thus how much accumulated evidence is required before making a decision. The boundary separation parameter reflects speed-accuracy tradeoffs, with individuals with narrow decision bounds making more ‘risky’ decisions, prioritising speed over accuracy, and individuals with wider decision bounds making more cautious decisions. The starting point can reflect bias, if the evidence accumulation process starts nearer to one of the bounds than the other. The non-decision time reflects sensory encoding and response generation that happen outside of the decision process, but contribute to the overall response time.

Compared to previous studies of motion processing in autism which focus purely on accuracy indices (psychophysical thresholds), the diffusion model approach offers a few advantages. First, it can help us address whether differences between autistic and non-autistic participants reflect differences in non-decisional processes (including sensory encoding and response generation), the ability to extract motion evidence (reflecting sensitivity to motion information) and/or decisional strategies (i.e., how much information is needed before committing to a decision), to provide a better understanding of *why* autistic individuals process sensory information differently to those without autism. Differences between autistic and non-autistic individuals could emerge at multiple and different stages of processing, consistent with the theoretical suggestion that we should forfeit a single mechanism underpinning autism^36,37^. Second, diffusion model parameters may be more sensitive to group differences than accuracy or response time alone^38,39^, meaning that we may be able to better detect differences between autistic and non-autistic participants and therefore better characterise group differences in sensory processing. Third, the resulting parameters have been linked to neural correlates^40^, meaning that they can give insights into neurobiologically plausible processing stages, thus linking levels of explanation from brain to behaviour.

The utility of this approach for understanding autistic perception has recently been shown in a study of orientation discrimination by Pirrone et al.^41^. In this study, autistic adults had longer response times, but similar accuracies, compared to non-autistic individuals. Diffusion modelling revealed that autistic participants responded more cautiously (i.e., had wider decision bounds) and had longer non-decision times than non-autistic individuals, yet the groups did not differ in terms of drift-rate, suggesting similar levels of sensitivity to orientation information. Without such modelling, it may have been concluded from autistic individual’s slower responses that they were less sensitive to orientation information. The finding of increased response caution was later replicated in a study of orientation discrimination in autistic children aged 6 to 16 years^42^, although in this study, there was no evidence for differences between autistic and typically developing children in non-decision time, and it was not possible to interpret any differences in drift-rates between the groups. This is because drift-rate not only reflects an individual’s sensitivity but also the stimulus strength, and in this study, the stimulus strength was tailored to each individual’s sensitivity, so that no group differences would be expected in drift-rate even if the groups differed in their underlying sensitivity.

Three further studies have used the diffusion model with autistic populations to understand stereotypical attitudes in an implicit association task^43^, performance in the correct ‘go’ trials of a response inhibition stop-task^44^ and numerical addition problems^45^. In all studies, autistic individuals were reported to display a more cautious response style than non-autistic individuals, in line with the findings of Pirrone et al.^41,42^. Conversely, none of the studies found evidence of differences in non-decision times between autistic and non-autistic individuals. Karalunas et al.^44^ reported that autistic individuals had reduced drift-rates compared to non-autistic individuals, c.f.^41^, but Kirchner et al.^43^ and Iuculano et al.^45^ found no group differences in this parameter. Combining the results of these studies together, it seems that differences in boundary separation between autistic and non-autistic individuals may be ubiquitous across the tasks tested, while differences in drift-rate and non-decision time might depend on the task and age of participants, as well as other differences between studies, such as analytical approach.

In this study, we applied the diffusion model approach in combination with EEG to better understand visual motion processing in autistic children. We used both a motion coherence task and a direction integration task because we expected them to reveal different effects on diffusion model parameters, due to their different requirements for segregating signal-from-noise. Specifically, the direction integration task does not require segregating signal dots from noise dots, unlike the motion coherence task. The use of both tasks therefore means we can ascertain whether differences in perceptual decision-making in autism are task-specific. We combined the diffusion model approach with electroencephalographic (EEG) data because both techniques investigate multiple processing stages and temporal dynamics that are not captured by simple accuracy indices and therefore have the potential to provide novel, multi-level insights into motion processing in autism (see Ref.^46^). A purely behavioural diffusion model analysis alone would have restricted our insights to the computational or algorithmic level of explanation, and therefore would not inform on the underlying implementation (cf. Ref.^47^). By contrast, our decision to combine diffusion modelling with EEG augments the insights that can be obtained, by linking computational and implementational levels of explanation and allowing us to comment on the biological plausibility of the computational stages identified. This aim is in line with the emerging field of model-based cognitive neuroscience (see Refs.^48,49^ for review).

We pre-registered four hypotheses prior to completing data collection and prior to data analysis (https://osf.io/znyw2):

1. Autistic children will have reduced drift-rates in the motion coherence task compared to typically developing children, in line with previous reports of reduced motion coherence sensitivity in autistic individuals, e.g.,^16,17,50^. This would suggest a reduced ability to extract visual evidence from the motion coherence task (which requires both integration over signal dots and segregation of signal dots from noise dots).
2. Autistic children will have increased drift-rates in the direction integration task compared to typically developing children, based on previous reports of enhanced discrimination performance in this task^18,23^. This would suggest an enhanced ability to extract visual evidence from the direction integration task (which requires integration over signal dots but no requirement to segregate signal dots from noise dots).
3. Autistic children will show wider response boundaries compared to typically developing children in both motion coherence and direction integration tasks^41–45^, suggesting a more cautious decision-making style in autistic participants.
4. Autistic children will show longer non-decision times than typically developing children in both motion coherence and direction integration tasks, following Pirrone et al.^41^. This would suggest that non-decisional processes such as sensory encoding and response generation take longer in autistic participants than non-autistic participants. Other studies have reported no differences in non-decision time between autistic and typical individuals^43,44^, but we reasoned that the visual discrimination task used by Pirrone et al. was more similar to our tasks. However, we note that a study comparing autistic and typically developing children on an orientation discrimination task was published since pre-registering our hypotheses showing no group differences in non-decision times^42^.

These hypotheses were addressed by models fit to the behavioural data. By collecting high-density EEG data from the majority of participants, we were also able to investigate links between the model parameters and EEG markers, to link levels of explanation^48,49^. Owing to its high temporal resolution, EEG provides potentially complementary insights into the stages of processing that might be affected in autism, allowing links to be made between brain and behaviour^51–53^. A response-locked neural marker of the decision-making process that correlates with drift-rate has been identified over centro-parietal electrodes in both typically developing children^31^ and children with dyslexia^54^, with children with dyslexia showing a reduced drift-rate compared to typically developing children, and corresponding reductions in the neural marker. We therefore expected that any differences in drift-rate would be related to response-locked activity over centro-parietal electrodes, thus providing a neural marker of atypical drift-rate.

## Methods

### Blind modelling and sampling plan

Due to difficulties in specifying all modelling decisions before seeing the data, we used a blind modelling approach^55^, with an author who was not involved in data collection (N.J.E.) acting as a blind modeller (see pre-registration document: https://osf.io/znyw2). The blind modeller made all modelling decisions (e.g., removal of outliers, specification of priors) in blinded datasets prepared by the first author (C.M.) in which the key variable relevant to the hypotheses under test (group membership) was randomly permuted.

While there is no established method for conducting power analyses for hierarchical Bayesian diffusion models, we conducted Monte Carlo simulations^56^ which suggested that 49 participants per group were required on average to detect a simple group difference with a moderate effect size of *d* = 0.5. We decided on an upper limit of 50 participants per group due to resource limitations. As we could not be certain of the expected effect size, we followed a 2-stage analysis plan to aim for confidence in our results while minimising unnecessary testing of participants. In the first stage, we analysed the data from groups of 40 autistic and 40 typically developing children. The first 40 participants in each group were not well-matched in performance IQ, so we tested another 10 typically developing children. We then selected the 40 typically developing children who best matched the 40 autistic children in age and performance IQ, using the R MatchIt package^57^. The blind modeller analysed the blinded dataset (analysis code here: https://osf.io/f3wnt/) and the first author then ran the analysis on the unblinded dataset. As we did not have strong evidence for or against our hypotheses (all Bayes factors (BF) between 1/6 and 6) at this stage, we proceeded to the second stage of our analysis plan. We resumed data collection until we had groups of 60 typically developing children and 50 autistic children (regardless of the level of evidence for or against our hypotheses). We selected the 50 typically developing children who best-matched the 50 autistic children in terms of age and performance IQ. The blind modeller again analysed the blinded dataset (analysis code here: https://osf.io/f3wnt/), before the final unblinded analyses were performed by the first author.

### Participants

Following the steps outlined in our sampling plan, our final dataset included 50 autistic children (40 males) and 50 typically developing children (27 males). All children included in the dataset had normal or corrected-to-normal acuity (6/9 or better for children aged 6–8 years and 6/6 or better for children aged 9–14 years), as measured by a Snellen acuity chart, and no evidence of intellectual disability (verbal IQ > 70 and performance IQ > 70 on the Wechsler Abbreviated Scales of Intelligence, 2nd edition [WASI-II^58^]). All typically developing children included in the dataset scored below the cut-off for autism (< 15) on the Social Communication Questionnaire (SCQ^59^) completed by parents. All autistic children included in the dataset had an independent clinical diagnosis of autism, and scored above the cut-off for an autism spectrum condition on the Social Communication Questionnaire (≥ 15) and/or on the Autism Diagnostic Observation Schedule, 2nd edition (ADOS-2^60^) (see Refs.^18,23^). Demographics for the participants included in the dataset are presented in Table 1. EEG data were collected during task performance in a subset of these children: in the motion coherence task we had EEG data for 46 typically developing and 43 autistic children and in the direction integration task we had EEG data for 45 typically developing and 45 autistic children. The EEG data from some of these participants has been included in a paper investigating responses locked to the onset of global motion in autistic and dyslexic children^22^. Datasets from an additional 2 typically developing children were excluded due to poor visual acuity (*n* = 1) or not meeting the criterion of correct responses in the practice phase (*n* = 1), and datasets from an additional 7 autistic children were excluded due to poor visual acuity (*n* = 1), having an IQ below 70 (*n* = 2) or not meeting the autism cut-offs in either the SCQ or ADOS (*n* = 4). Many of the typically developing children overlapped with those in the control group in a related study of dyslexia^54^. As we expected a relationship between dyslexia and perceptual decision-making^61^, we measured reading ability with the Test of Word Reading Efficiency—2nd Edition (TOWRE-2^62^) and measured spelling ability using the Wechsler Individual Achievement Test (WIAT-III^63^) in all participants (see Table 1 for standard scores). As decision-making parameters have also been shown to diverge in children with inattentive and hyperactive symptoms^44,64^, we also collected parent-report ratings on 9 items assessing children’s inattention and 9 items assessing hyperactivity/impulsivity, from the DSM-IV^65^ criteria for ADHD as used in the Swanson, Nolan and Pelham-IV Questionnaire (SNAP-IV^66^; https://www.amerihealth.com/pdfs/providers/resources/worksheets/prevhealth_swan.pdf) to characterise the sample and allow exploratory analyses between ADHD symptoms and decision-making parameters. These items were binary coded and an average item score was extracted for each of the inattention and hyperactivity/impulsivity dimensions. As shown in Table 1, the autistic children had higher scores than typically developing children on both dimensions.

**Table 1:** Demographics of participants included in final dataset.

	Typically developing (n = 50)	Autistic (n = 50)
Age	10.61 (2.34) 6.55–14.98	10.91 (2.43) 6.54–14.94
Performance IQ	110.44 (13.00) 81–145	104.98 (14.99) 78–138
Verbal IQ	110.90 (9.64) 95–142	104.28 (14.86) 76–137
Full-scale IQ	112.06 (10.49) 89–142	105.22 (15.09) 76–133
SCQ	2.46 (2.44) 0–11	20.45 (7.12) 6–36
ADOS-2—total		12.14 (5.30) 3–27
WIAT III spelling	110.42 (16.27) 81–146	94.70 (22.81) 50–152
TOWRE-2: phonemic decoding efficiency	105.02 (10.17) 80–135	96.96 (18.69) 55–132
Inattentiveness	0.17 (0.23) 0–1	0.58 (0.29) 0–1
Hyperactivity/impulsivity	0.08 (0.19) 0–1	0.55 (0.34) 0–1

Data are presented as M (SD) Range.

*SCQ* Social Communication Questionnaire^59^, *ADOS-2* Autism Diagnostic observation schedule, 2nd Edition^60^, *WIAT-III* Wechsler Individual Achievement Test, 3rd Edition^63^, *TOWRE-2* Test of Word Reading Efficiency, 2nd edition^62^. Standard scores are presented for the WIAT-III and TOWRE-2. Average item scores are presented for the Inattentiveness and Hyperactivity/impulsivity scales.

### Apparatus

Stimuli were presented on a Dell Precision M3800 laptop (2048 × 1152 pixels, 60 Hz) using the Psychophysics Toolbox for MATLAB^67,68^. EEG signals were collected with 128-channel Hydrocel Geodesic Sensor Nets connected to Net Amps 300 (Electrical Geodesics Inc., OR, USA) and NetStation 4.5 software. A photodiode attached to the monitor independently verified the timing of stimulus presentation. Participants provided responses on a Cedrus RB-540 response box (Cedrus, CA, USA).

### Stimuli

**Figure 2:**
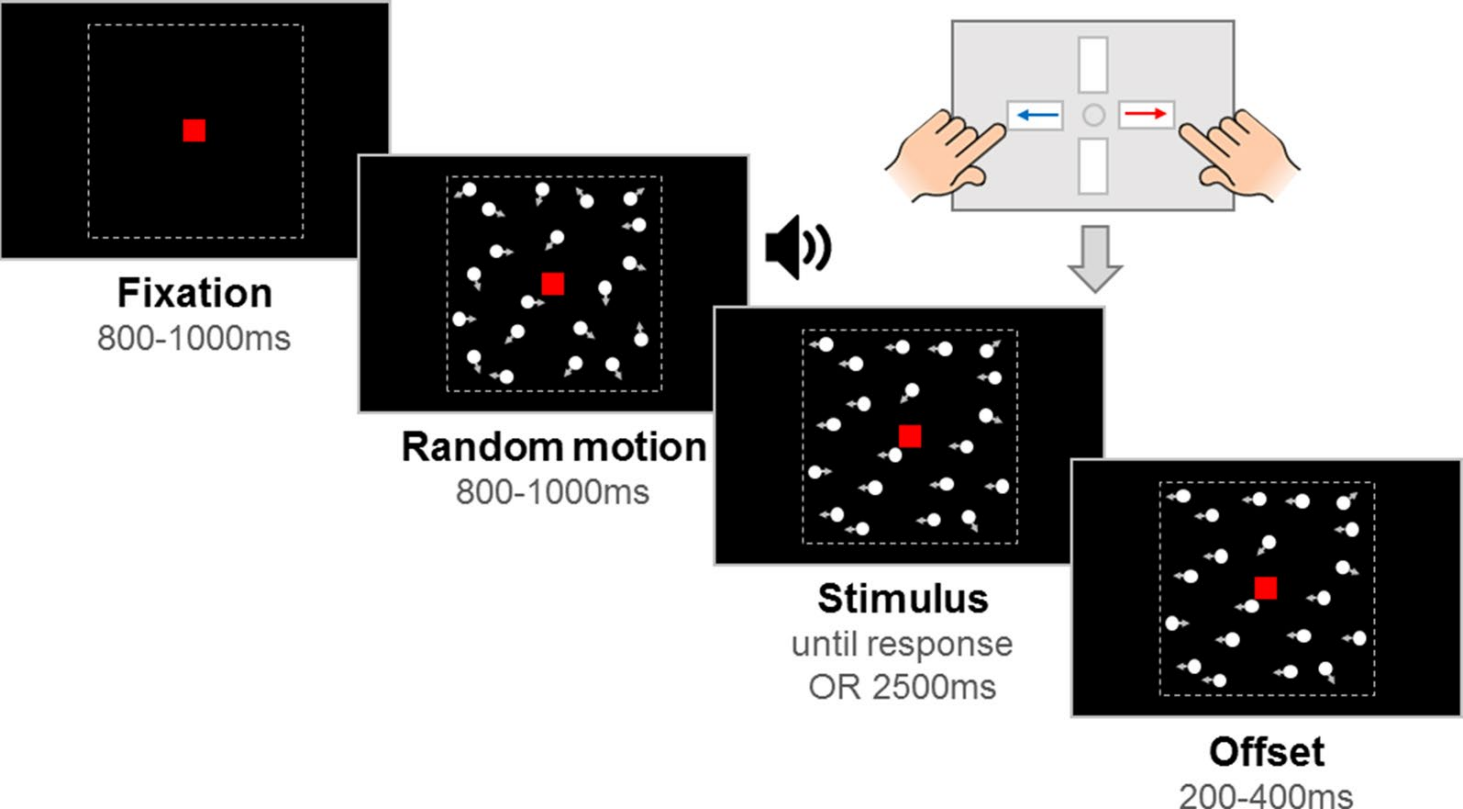
Schematic representation of trial procedure. An initial *fixation* period was followed by a *random motion* period consisting of randomly moving dots. Next, the *stimulus* period contained leftward or rightward global motion and lasted until the child reported the direction with a response box. If there was no response, the stimulus remained on the screen for 2500 ms. The stimulus remained on the screen for an *offset* period after the response or after the maximum stimulus duration was reached. Note that arrows (indicating movement) and dotted lines (marking the square stimulus region) are shown for illustrative purposes only. Figure reproduced from https://osf.io/wmtpx/ under a CC-BY4.0 license.

Stimuli comprised of 100 white, randomly positioned dots (diameter 0.19°) moving inside a square aperture (10° × 10°) at 6°/s on a black screen. The dots had a limited lifetime of 400 ms (following a randomly assigned starting life), at which point they were randomly replotted in a new location. A red fixation square (0.24° × 0.24°) was presented throughout the trial. Each trial consisted of a fixation period, a random motion period, a stimulus period, and an offset period (see Fig. 2). The fixation period had a randomly selected duration between 800 and 1000 ms. The random motion period presented the stimulus dots moving in random, incoherent directions, for a randomly selected duration between 800 and 1000 ms. The random motion period ensured that pattern- and motion-onset evoked potentials were distinguished temporally from the onset of directional motion in the stimulus period. However, the presentation of the random motion period meant that there was temporal uncertainty about when the stimulus period began, with implications for the modelling^31^. Therefore, at the start of the stimulus period, we presented an auditory tone to signal to participants that the directional motion had started. Note that the stimulus onset could still not be overly anticipated due to the jitter in stimulus onset (between 800 and 1000 ms after the random motion period began). In the motion coherence task, directional motion (leftward or rightward) was introduced in a proportion of ‘signal’ dots, while the remainder of the dots continued to move in random directions. In the direction integration task, the directions of dots in the stimulus phase were distributed according to a Gaussian distribution with a mean leftward or rightward direction. The stimulus period was presented until a response was made, or until 2500 ms had elapsed. Finally, the offset period continued the directional motion for a randomly selected duration between 200 and 400 ms, which served to dissociate response-locked EEG activity from that associated with motion offset.

### Experimental task procedure

Children were presented with both motion coherence and direction integration tasks—the order of which was counterbalanced between participants. The tasks were presented in the context of a child-friendly game set in ‘Insectland’ (based on Ref.^31^). Using animations, it was explained to participants that the fireflies were escaping from their viewing boxes, and that the zookeeper needed to know through which side of the box the fireflies were escaping, so that he could fix the box and ‘save’ the fireflies. Participants were told that there were 10 different viewing boxes, corresponding to 10 different ‘levels’ of the game. Levels 1–5 corresponded to one task (either motion coherence or direction integration), and Levels 6–10 corresponded to the other task, depending on the counterbalancing order for each participant. Levels 1 and 6 were practice phases, and the experimental blocks were presented in the remaining 4 levels for each task. In the motion coherence task, difficulty was manipulated by varying the proportion of dots moving coherently, and in the direction integration task, difficulty was manipulated by varying the standard deviation of the distribution from which the dot directions were sampled.

The practice phases began with four demonstration trials with no random motion phase and an unlimited stimulus phase, to introduce the task to participants. Participants used the 'left’ and ‘right’ arrow keys on the response box to report the direction. The first two demonstration trials were designed to be ‘easy’ (100% coherence or 1° standard deviation), while the last two were more difficult (75% and 50% coherence, or 10° and 25° standard deviations). Next, there were up to 20 criterion trials (coherence of 95% or 5° standard deviation), which included the random motion period. It was explained to participants that the fireflies would be going “all over the place” at first, so that they must wait for the fireflies to escape before responding. They were told that there would be an alarm (auditory beep) to help them, and that they must wait for this alarm before deciding which way the fireflies were escaping. This auditory cue removed temporal uncertainty about the onset of the stimulus phase. There was a time limit of 2500 ms, with visual feedback presented if participants did not respond within this time (“Timeout! Try to be quicker next time!”, presented in red text). When participants met a criterion of four consecutive correct responses, they moved on to eight practice trials which increased in difficulty (motion coherence task: 80%, 70%, 60%, 50%, 40%, 30%, 20%, 10%; direction integration task: 5°, 10°, 15°, 20°, 30°, 40°, 50°, 60°). Participants were reassured that it was fine if they got some of these wrong and/or if they had to guess. Visual feedback was presented after each trial throughout Level 1 (“That was correct!” presented in green text, or “It was the other way that time” or “Timeout! Try to be quicker next time!”, presented in red text). For 1 child, Level 1 was repeated as they did not meet the criterion of four consecutive correct responses on the first attempt but did on the second.

Levels 2–5 and 7–10 each consisted of 38 trials (totalling 152 trials for each task), with 9 repetitions for each of two difficulty levels (motion coherence task: 30%, 75%; direction integration task: 70°, 30° SD) and each motion direction (leftward, rightward), and 2 catch trials presenting 100% coherent (0° SD) motion. We used the same difficulty levels for all participants (rather than tailoring them to each individual’s sensitivity) so that we could interpret any group differences in drift-rate as reflecting differences in sensitivity. The difficulty levels were chosen through piloting, to ensure that children of all ages would be able to complete the task, while still obtaining enough errors for diffusion modelling. No trial-by-trial feedback was presented in the experimental phase, apart from a ‘timeout’ message (as before) if no response was given within 2500 ms after the stimulus period began. At the end of each level, participants were presented with their ‘points’ for the preceding block of trials. Participants were told that they would get points for both speed and accuracy. The points reflected an efficiency score, computed by (1 / median RT) * number of correct responses * 2, rounded to the nearest integer. In the event that participants obtained a score under 10, a score of 10 points was given to avoid demotivation. Trials were presented automatically, but the experimenter had controls to pause and resume trial presentation (see below). See https://osf.io/f3wnt/ for experimental code.

### General procedure

Ethical approval was provided by the Central University Research Ethics Committee at the University of Oxford (R56348/RE004) and the study was conducted in accordance with this approval. Parents/carers of child participants provided written informed consent, and children provided verbal or written assent. Most children took part at the University of Oxford, although one child took part at home without EEG. Participants initially completed a Snellen acuity test to confirm normal or corrected-to-normal vision. During the experimental tasks, participants sat 80 cm away from the computer screen in a dimly lit room. For children who were willing to take part with EEG, we fitted the net prior to the experiment and ensured that electrode impedances were below 50 kΩ. EEG data were acquired at a sampling rate of 500 Hz with a vertex reference.

Children were monitored by an experimenter during the tasks. The experimenter gave regular encouragement and task reminders, pausing before starting a trial where necessary (e.g., to remind the child to stay still). Children had short breaks at the end of each block (or ‘level’), and a longer break after completing the first task (i.e., at the end of ‘level 5’). The electrode impedances were re-assessed for children wearing EEG nets during this longer break. Children recorded their progress through the levels using a stamper and a record card. The children also completed the WASI-II, TOWRE-2 and the WIAT-III spelling subtest. The autistic children also completed module 3 or 4 of the ADOS-2. The whole session took no longer than 2 h for typically developing children and no longer than 3 h for autistic children. Children were given a £10 voucher to thank them for their time.

### Drift–diffusion modelling of data

Trials with RTs under 200 ms were removed (0.20% of trials in the typically developing group and 0.26% of trials in the autism group). Trials where no response was made within 2500 ms were modelled as non-terminating accumulation trajectories, where the probability of a non-response occurring was the survivor function for the model at the time of the 2500 ms deadline^69–71^. These trials accounted for 1.01% of the data in the typical group and 0.95% of the data in the autism group. We fit the data from each task with hierarchical, Bayesian diffusion models with 5 parameters: (1) average drift-rate across difficulty levels *v.mean,* (2) boundary separation *a*, (3) non-decision time *ter*, (4) difference in mean drift-rate between difficulty levels *v.diff*, and (5) starting point *z.* The stochastic noise in the model (*s*) was fixed at 0.1 to solve a scaling problem within the model, following convention^33^. There were 3 hyperparameters for each parameter reflecting the mean (µ) and standard deviation (σ) across the two groups and the difference between groups (δ). This parameterization meant we could explicitly set priors on the differences between groups, which relates to our main hypotheses. Specifically, the priors were:

Data level:

$${y}_{pi} \sim diffusion\left({a}_{p, } {z}_{p},{Ter}_{p},{v}_{pi}, s\right)$$

Parameters:

$${a}_{p} \sim {N}_{+}\left({\mu }_{a} \pm {\delta }_{a}, {\sigma }_{a}\right)$$

$${z}_{p}/{a}_{p} \sim {TN}_{\mathrm{0,1}}\left({\mu }_{z} \pm {\delta }_{z}, {\sigma }_{z}\right)$$

$${Ter}_{p} \sim {N}_{+}\left({\mu }_{Ter} \pm {\delta }_{Ter}, {\sigma }_{Ter}\right)$$

$${v}_{p1}-{v}_{p2} \sim N\left({\mu }_{v.diff} \pm {\delta }_{v.diff}, {\sigma }_{v.diff}\right)$$

$$\frac{{v}_{p1}+ {v}_{p2}}{2}\sim N\left({\mu }_{v.mean} \pm {\delta }_{v.mean}, {\sigma }_{v.mean}\right)$$

$$s=0.1$$

Hyperparameters:

$${\mu }_{a} \sim {N}_{+}\left(0.2, 0.2\right)$$

$${\mu }_{z} \sim {TN}_{\mathrm{0,1}}\left(0.5, 0.2\right)$$

$${\mu }_{Ter} \sim {N}_{+}\left(0.3, 0.3\right)$$

$${\mu }_{v.diff} \sim N\left(0, 0.1\right)$$

$${\mu }_{v.mean} \sim N\left(0.3, 0.3\right)$$

$${\sigma }_{a},{\sigma }_{z},{\sigma }_{Ter},{\sigma }_{v.diff},{\sigma }_{v.mean} \sim \Gamma \left(1, 1\right)$$

$${\delta }_{a},{\delta }_{z},{\delta }_{Ter},{\delta }_{v.diff},{\delta }_{v.mean} \sim N\left(0, 0.01\right)$$

where *y* reflects the data, and subscripts *p* and *i* reflect the participant and difficulty level respectively. The priors for the *µ* and *σ* parameters were based on previous studies applying hierarchical diffusion models^72–74^, and the priors for the *δ* parameters were derived from the “moderately informative priors” used for condition differences in Ref.^75^. We used a differential evolution Markov chain Monte Carlo algorithm (DE-MCMC^76,77^) to sample from the posterior with 15 interacting chains. Each chain had 4000 iterations, with the first 1500 discarded as burn-in. We also used a migration algorithm^77^, in which chains were randomly migrated every 14 iterations between iterations 500 and 1100.

Bayesian t-tests using the BayesFactor R package^78^ showed that the final groups (n = 50 per group) were similar in age (Bayes factors [BF] in support of group differences = 0.25), but there was weak, anecdotal evidence (1/3 < BF < 3) for group differences in performance IQ (BF = 1.12). The blind modeller therefore constructed models with and without the effects of performance IQ partialled out, so that we could assess the effect that this had on the results. The first author (CM) then conducted the unblinded analysis with correct group membership. In light of a previous study finding reduced drift-rate in children with dyslexia^54^, we also assessed group differences in an exploratory model which partialled out reading and spelling ability.

### EEG preprocessing and analyses

EEG data were band-pass filtered between 0.3 and 40 Hz using NetStation filters before being further processed in MATLAB with EEGLAB functions^79^. We downsampled each participant’s data to 250 Hz and extracted only the data between the first fixation onset and the last offset period. We again bandpass-filtered between 0.3 and 40 Hz (due to insufficient attenuation of low frequencies by NetStation filters^80^) and used EEGLAB’s clean_artifacts function to remove bad channels and identify data segments with standard deviations over 15 and correct them with artifact subspace reconstruction^81^. Missing channels were then interpolated. We then ran independent components analysis on 3000 ms epochs starting at fixation onset using an Infomax algorithm and subtracted ocular components from the continuous data. Finally we re-referenced the data to an average reference.

**Figure 3:**
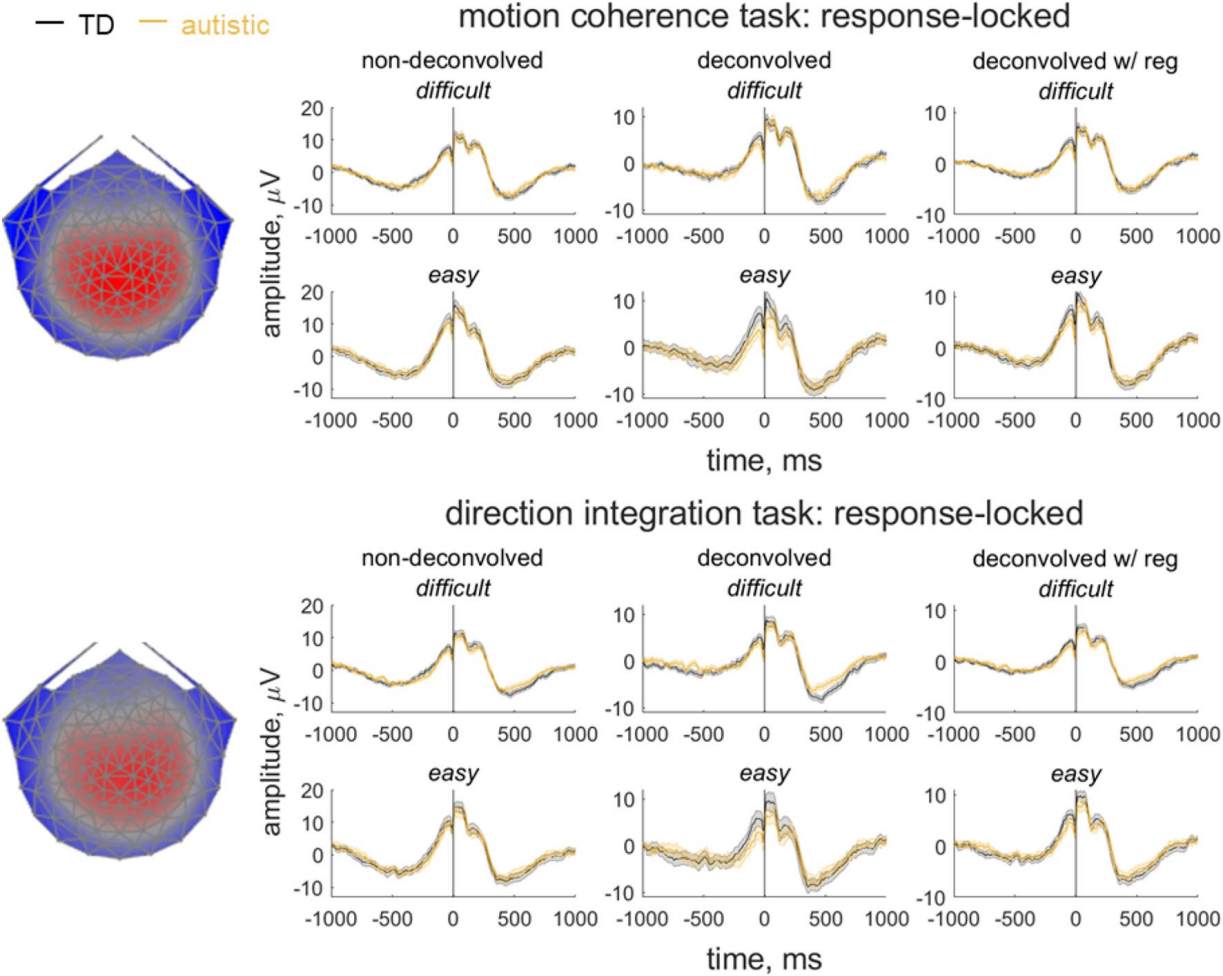
Response-locked evoked potentials for the motion coherence task and direction integration task. Topographic maps represent the forward-model projections of the most reliable component reflecting the weights given to each electrode following reliable components analysis (RCA) on data from all participants pooled across difficulty level for the motion coherence task (top) and direction integration task (bottom). Each individual’s continuous data were multiplied by these weights to provide a component average waveform, with group average response-locked waveforms (± 1SEM) shown for typically developing children (TD; grey) and autistic children (orange) for difficult and easy levels. The left column shows non-deconvolved group average waveforms. The central column shows deconvolved group average waveforms (without regularisation). The right column shows deconvolved group average waveforms with regularisation (ridge regression).

We used a data-driven component decomposition technique, Reliable Components Analysis^82,83^, to identify spatiotemporally reliable patterns of activity across trials, for integration with our diffusion model. First, we epoched each participant’s preprocessed continuous data from 600 ms before the response to 200 ms after the response, for all responses made between 200 and 2500 ms after stimulus onset. We baselined the data to the last 100 ms of the random motion period and submitted the baselined epochs for participants in both groups to Reliable Components analysis for each task separately. The forward-model projection of the weights for the most reliable component for each task is shown in Fig. 3. This component explained 33.7% and 30.5% of the trial-to-trial covariance in the motion coherence and direction integration tasks, respectively. This component resembled the most reliable component found in our previous work^31,54^, which resembles the centro-parietal positivity^29,30^ and has been linked to drift-rate in typically developing children^31^ and children with dyslexia^54^. To investigate links with drift-rate in this dataset, we multiplied each participant’s continuous data with the spatial weights for this component to yield a single component average waveform for each participant.

In our paradigm, stimulus-locked and response-locked activity will overlap temporally, especially for short response times. Therefore, in order to extract a response-locked measure for inclusion in the diffusion model, we used a linear deconvolution method to unmix the overlapping stimulus-locked and response-locked activity using the Unfold toolbox^84^. We modelled the projected continuous data for each participant by selecting a time window from 1000 ms before to 1000 ms after each stimulus event or response event made between 200 and 2500 ms following stimulus onset. We specified a design matrix with predictors for each difficulty level (difficult, easy) and each event type (stimulus, response). Next we time-expanded the design matrix by adding a predictor for each timepoint sampled for each event type and excluded segments with amplitudes above 250 mV from the design matrix (motion coherence task: mean 2.72% of the data for each participant, range: 0–43.49%; direction integration task: mean = 3.00%, range: 0–49.30%). We then fit the deconvolution model resulting in regression weights (betas) for each of the 2 difficulty levels, 2 event types and 500 timepoints, which we used to construct regression ERP waveforms. The response-locked waveforms are presented in Fig. 3 and stimulus-locked waveforms are presented in Supplementary Fig. S1. Additionally, as a complement to previous analyses^35^, Supplementary Fig. S2 shows stimulus-locked waveforms relating to the second-most reliable component, previously linked to motion-specific processing^22^. While not the focus of the current investigation, this figure suggests that the groups were similar in their early encoding of motion evidence (up to ~ 400 ms following stimulus onset, see also Ref.^22^).

While the non-deconvolved waveforms showed the expected amplitude differences between the two difficulty levels, these differences were not apparent in the deconvolved waveforms. As the difference between difficulty levels changed following deconvolution, we considered the possibility that the overlap between stimulus- and response-locked activity differed between the difficulty levels, due to different RT distributions in each difficulty level. However, the non-deconvolved waveforms showed a difference between difficulty levels even when the RT distributions were matched across the two levels, suggesting that such differences cannot be solely attributed to different RT distributions. Instead, we suspected that the beta estimates were noisy and that the deconvolution technique was overfitting the noise. Therefore, in the final step where we extracted EEG measures for inclusion in the diffusion model, we re-ran the deconvolution model using a ridge-regression regularisation method which penalises the squared magnitude of the regression coefficients (see Ref.^85^) to minimise noise. Accordingly, the difficulty level differences were preserved when applying deconvolution with ridge-regression regularisation. We found the best regularisation parameter for each participant using cross-validation, and then took the mode across all participants and constrained the regularisation parameter to ensure that differences in regularisation did not contribute to group differences in resulting waveforms. The modal parameter value was 4.5 for the motion coherence task (5.5 and 6 for the autistic and typically developing children, separately) and 5.5 for the direction integration task (5.5 in both groups separately). We then fit a linear regression slope to each participant’s average deconvolved waveform for each difficulty level from 200 ms before the response to the time of the response, to obtain a slope measure which we entered into the joint diffusion model and related to drift-rate. We investigated group differences in this slope measure using a Bayesian repeated measures ANOVA using JASP^86^, with difficulty level as a within-participants factor and group as a between-participants factor.

### Joint modelling: EEG

To assess the relationship between drift-rate and the EEG measure discussed above, we used a joint modelling approach^69,87–90^. We estimated additional hyper-parameters for the correlation between the *v.mean* parameter and the average of the EEG measure over difficulty levels *(EEG.mean)*, and between the *v.diff* parameter and the difference in the EEG measure between difficulty levels *(EEG.diff)*. This meant that the structure of the original hierarchical model (with age partialled out) only differed for the drift-rate parameter, which was now a bivariate normal with the EEG measure:

$$\genfrac{}{}{0pt}{}{\left[{v}_{p1}-{v}_{p2}, {EEG}_{p1}-{EEG}_{p2}\right] \sim }{BN\left(\left[{\mu }_{v.diff}\pm {\delta }_{v.diff},{\mu }_{EEG.diff}\pm {\delta }_{EEG.diff}\right],\left[{\sigma }_{v.diff}^{2},{\sigma }_{v.diff}{\sigma }_{EEG.diff}{\varvec{\rho}},{\sigma }_{EEG.diff}{\sigma }_{v.diff}{\varvec{\rho}},{\sigma }_{EEG.diff}^{2}\right]\right)}$$

$$\genfrac{}{}{0pt}{}{\left[({v}_{p1}+{v}_{p2})/2, {(EEG}_{p1}+{EEG}_{p2})/2\right] \sim }{BN\left(\left[{\mu }_{v.mean}\pm {\delta }_{v.mean},{\mu }_{EEG.mean}\pm {\delta }_{EEG.mean}\right],\left[{\sigma }_{v.mean}^{2},{\sigma }_{v.mean}{\sigma }_{EEG.mean}{\varvec{\rho}},{\sigma }_{EEG.mean}{\sigma }_{v.mean}{\varvec{\rho}},{\sigma }_{EEG.mean}^{2}\right]\right)}$$

$${\mu }_{EEG.diff }\sim N\left(\mathrm{0,0.5}\right)$$

$${\mu }_{EEG.mean} \sim N\left(\mathrm{0,1}\right)$$

$${\sigma }_{EEG.diff},{\sigma }_{EEG.mean} \sim \Gamma \left(\mathrm{1,1}\right)$$

$${\delta }_{EEG.diff},{\delta }_{EEG.mean} \sim N\left(\mathrm{0,0.01}\right)$$

$${\varvec{\rho}}\sim U\left(-\mathrm{1,1}\right)$$

where ***ρ*** refers to the correlation between drift-rate and the EEG measure. We again used DE-MCMC with 15 interacting chains to sample from the posterior of the joint model, though here we used only 3000 iterations, with the first 1000 discarded as burn-in and no migration algorithm, as the joint model was more computationally intensive. We estimated two variants of this joint model: one where the correlations were constrained to be the same across groups, which would allow for the estimation of more precise posteriors, and another less constrained version where the correlations were estimated separately for each group. While we did not find evidence for group differences in age, we partialled out age in this joint model, because both the EEG parameter and drift-rate are known to change with age^31^. This ensured that any correlations identified between the EEG parameter and drift-rate did not merely reflect age-related changes.

### Joint modelling: ADHD

As the two groups differed in terms of ADHD symptoms, and in recognition of the literature on decision-making differences in children with inattention and hyperactivity symptoms^44,64^, we ran further, exploratory joint models to investigate the relationship between levels of parent-reported ADHD symptoms (for inattention and hyperactivity/impulsivity domains) and diffusion model parameters (note we did not pre-register any hypotheses for these relationships). We ran separate models which each correlated parent-reported ADHD symptoms with a different diffusion model parameter of interest, and we estimated the correlations separately for each group.

The priors for the ADHD joint model were near identical to the EEG joint model, though for the ADHD joint model (1) there were 5 different models, covering each parameter that could correlate with ADHD, (2) there were 2 ADHD subscales to assess (*inatt, hyper*), and (3) these subscales obviously did not vary between difficulty levels, meaning that there were no mean and difference values. The priors for the boundary separation-ADHD joint model can be seen below, with all other ADHD joint models following the same format:

$$\left[a, inatt\right]\sim BN\left(\left[{\mu }_{a}\pm {\delta }_{a},{\mu }_{inatt}\pm {\delta }_{inatt}\right],\left[{\sigma }_{a}^{2},{\sigma }_{a}{\sigma }_{inatt}{\varvec{\rho}},{\sigma }_{inatt}{\sigma }_{a}{\varvec{\rho}},{\sigma }_{inatt}^{2}\right]\right)$$

$$\left[a, hyper\right]\sim BN\left(\left[{\mu }_{a}\pm {\delta }_{a},{\mu }_{hyper}\pm {\delta }_{hyper}\right],\left[{\sigma }_{a}^{2},{\sigma }_{a}{\sigma }_{hyper}{\varvec{\rho}},{\sigma }_{hyper}{\sigma }_{a}{\varvec{\rho}},{\sigma }_{hyper}^{2}\right]\right)$$

$${\mu }_{inatt }\sim N\left(0.5, 0.2\right)$$

$${\mu }_{hyper }\sim N\left(0.5, 0.2\right)$$

$${\sigma }_{inatt},{\sigma }_{hyper} \sim \Gamma \left(\mathrm{1,1}\right)$$

$${\delta }_{inatt},{\delta }_{hyper} \sim N\left(\mathrm{0,0.01}\right)$$

$${\varvec{\rho}}\sim U\left(-\mathrm{1,1}\right)$$

## Results

### Accuracy and response time

**Figure 4:**
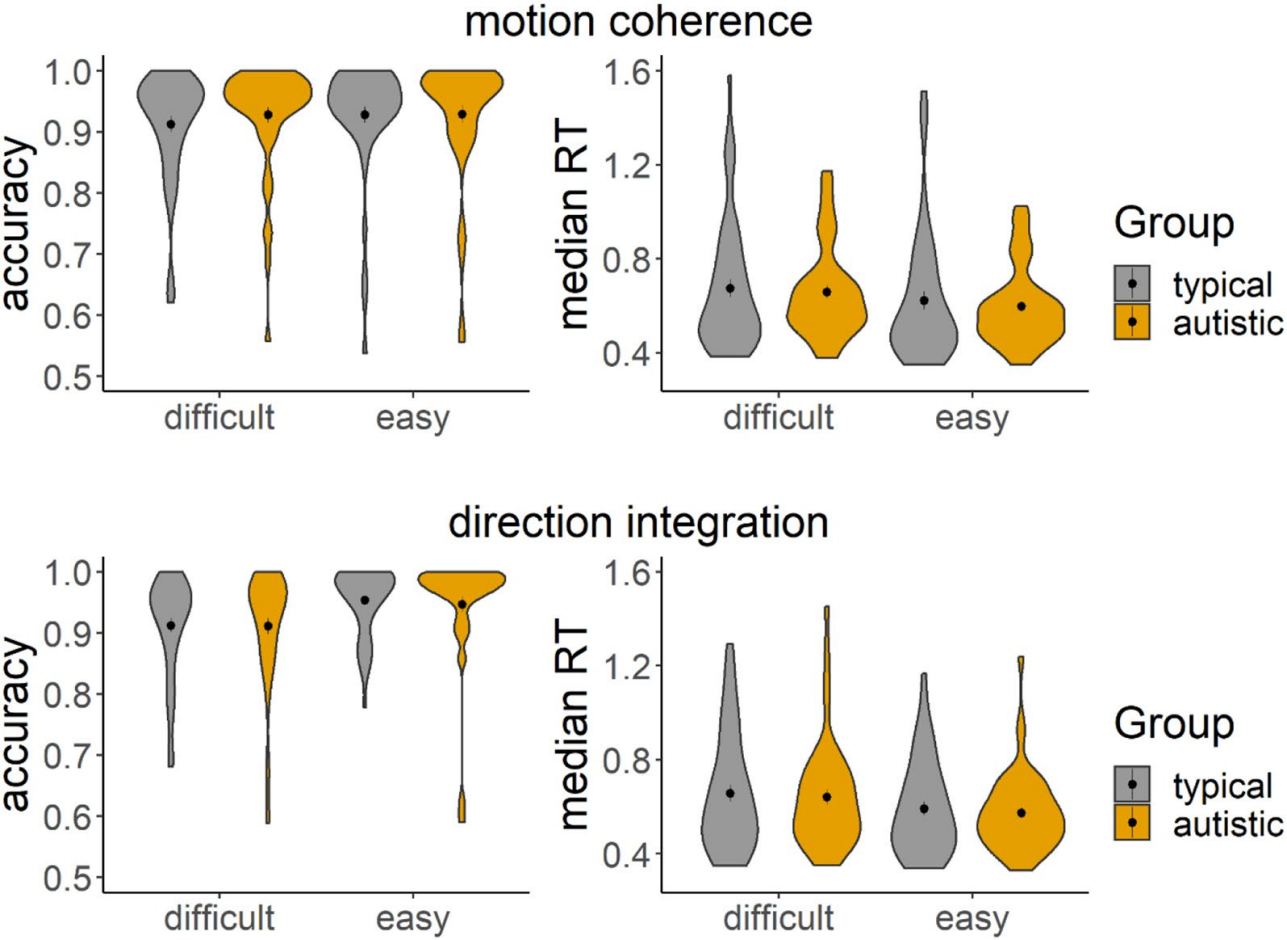
Accuracy and median response time (RT) for correct trials. Violin plots showing the kernel probability density for each group’s accuracy (left) and median RT (in seconds) for correct trials (right) for each difficulty level and each task (upper: motion coherence; lower: direction integration). The coloured ‘violins’ reflect the distribution of each group’s data points, with typically developing children and autistic children presented in grey and orange, respectively. Dots and vertical lines represent the group mean and ± 1 SEM.

The groups performed similarly in terms of overall accuracy and median RT, as shown in Fig. 4, with considerable between-participants variability. Bayesian mixed ANOVAs conducted in JASP^91^ with default priors showed relatively more evidence for no group differences than for group differences in accuracy and median RT. Specifically, the Bayes factors for including group in the model (BFincl) for accuracy were 0.30 and 0.32 in the motion coherence and direction integration tasks, respectively. BFincl for median RT were 0.60 and 0.42 in the motion coherence and direction integration tasks, respectively. BFincl for group x difficulty interaction terms were also all under 1.

### Diffusion modelling of behavioural data

**Figure 5:**
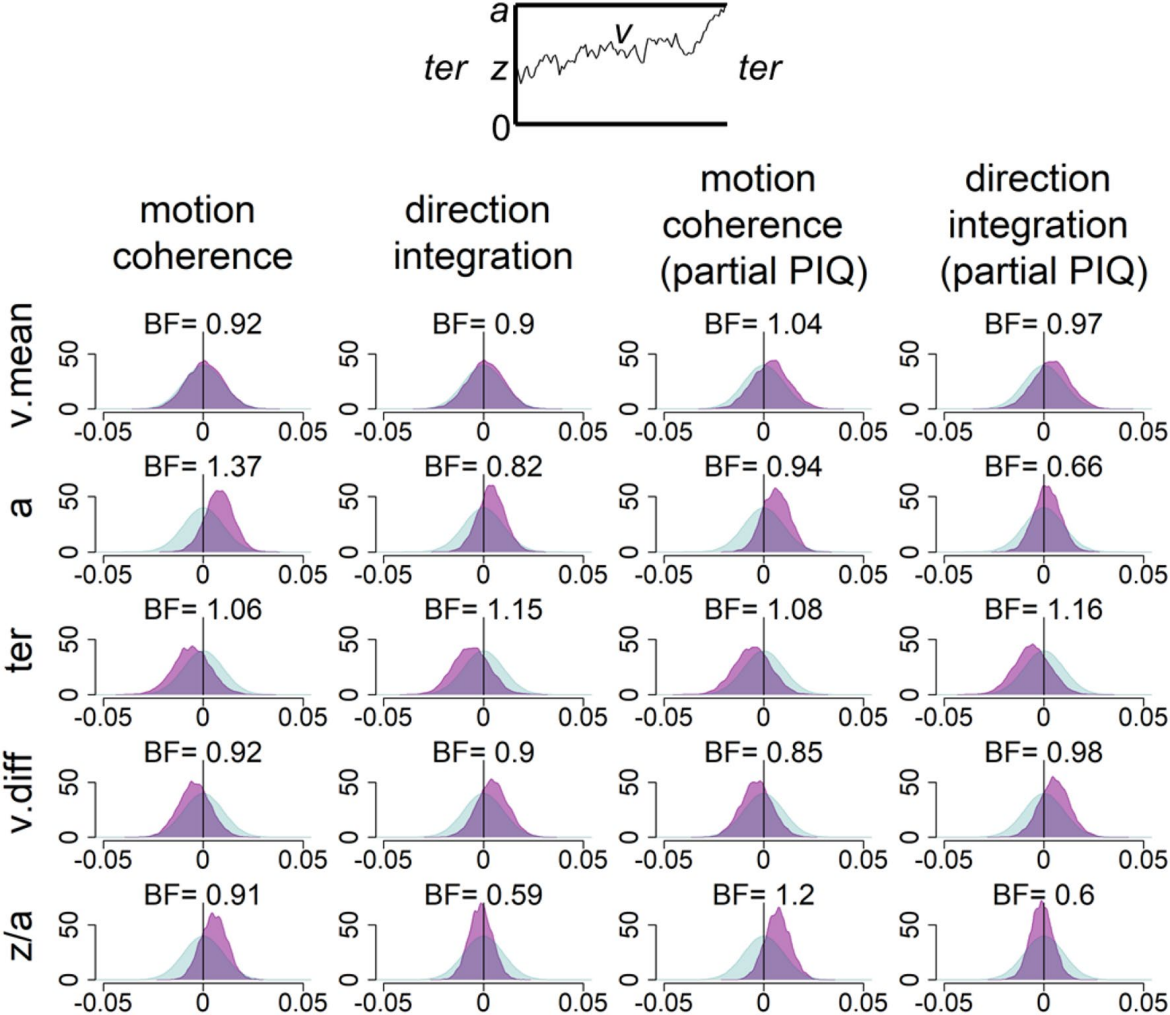
Prior and posterior density distributions. Prior (blue) and posterior (purple) density distributions for the group-level parameters reflecting group differences in each of the 5 model parameters (v.mean = mean drift-rate across difficulty levels; a = boundary separation; ter = non-decision time; v.diff = difference in mean drift-rate between difficulty levels; z/a = relative starting point) for each task. Inset is a schematic representation of the model parameters. Columns 1 and 2 show the results of the standard model and Columns 3 and 4 show the results of the model with performance IQ partialled out. Positive values reflect higher parameter values in the autistic group compared to the typically developing group. BF = Savage-Dickey Bayes factors in favour of the alternative hypothesis over the null hypothesis, where BF > 1 reflect more support for the alternative hypothesis.

Gelman-Rubin diagnostic values^92^ for all chains in all models were under 1.1 (mean = 1.00, range: 1.00–1.09), reflecting good convergence. To check model fit, we plotted the defective cumulative density functions for each group (Supplementary Fig. S3). Figure 5 shows the prior and posterior distributions for the group-level parameters that reflect the difference between groups for each of the 5 parameters (*v.mean, a, ter, v.diff, z/a*), along with Bayes factors calculated through the Savage–Dickey ratio. Bayes factors above 1 reflect more evidence for the alternative hypothesis of group differences compared to the null hypothesis, whereas Bayes factors below 1 reflect relatively more evidence for the null hypothesis than the alternative hypothesis. We interpret Bayes factors above 3 to reflect ‘moderate’ evidence for the alternative hypothesis and Bayes factors below 1/3 to reflect ‘moderate’ evidence in support of the null hypothesis, according to existing conventions^93,94^. Conversely, we interpret Bayes factors between 1/3 and 3 to represent weak, ‘anecdotal’ (i.e., inconclusive) evidence^93,94^.

As shown in Fig. 5, all Bayes factors were between 1/3 and 3, reflecting anecdotal (or inconclusive) evidence for our hypotheses. The posterior distributions were close to the prior distributions, suggesting that the data were relatively uninformative. Specifically, we had hypothesised that autistic children would have *reduced* drift-rates in the motion coherence task and *increased* drift-rates in the direction integration task, compared to typically developing children. Figure 5 suggests that there was little difference between groups in mean drift-rate in the standard model (as the posterior distribution for *v.mean* is centred close to 0), with weak, anecdotal evidence in favour of the null hypothesis (BF = 0.92 and BF = 0.9 for the motion coherence and direction integration tasks, respectively). When partialling out performance IQ there were very small group differences reflecting increased drift-rate in autistic children in both tasks (shown by the small rightward shift in the posterior distribution for *v.mean*), but the Bayes factors were very close to 1 (BF = 1.04 and BF = 0.97), suggesting near-equivocal evidence for the alternative and null hypotheses. We also hypothesised that autistic children would show wider response boundaries compared to typically developing children, and while the group difference was in the expected direction (shown by a rightward shift of the posterior distribution for *a*), the Bayes factors were again very close to 1 in the standard model (BF = 1.37 and BF = 0.82), and the evidence in support of the alternative hypothesis reduced slightly when controlling for the effects of performance IQ (BF = 0.94 and BF = 0.66). Finally, we hypothesised that autistic children would show longer non-decision times than typically developing children. Instead, the autistic children had slightly shorter non-decision times in both tasks (shown by a leftward shift of the posterior distribution for *ter*), but the Bayes factors were once again very close to 1, both in the standard model (BF = 1.06, BF = 1.15) and when controlling for performance IQ (BF = 1.08, BF = 1.16). These results suggest that more data would be required to provide conclusive evidence to support or refute our hypotheses.

In a related study of dyslexia, we found that children with dyslexia had *reduced* drift-rates compared to typically developing children^54^. Given that the autistic children had lower reading and spelling abilities than typically developing children, overall (Table 1), we conducted a follow-up analysis to ensure that the pattern of results did not change when controlling for children’s reading ability (assessed using the TOWRE-2 Phonemic Decoding Efficiency subscore) and spelling ability, in addition to performance IQ (see Supplementary Fig. S4). While some of the Bayes factors increased slightly, there remained no conclusive evidence for any of our hypotheses, with all Bayes factors being between 1/3 and 3 (i.e., in the ‘anecdotal’ range).

### Joint model: EEG

**Figure 6:**
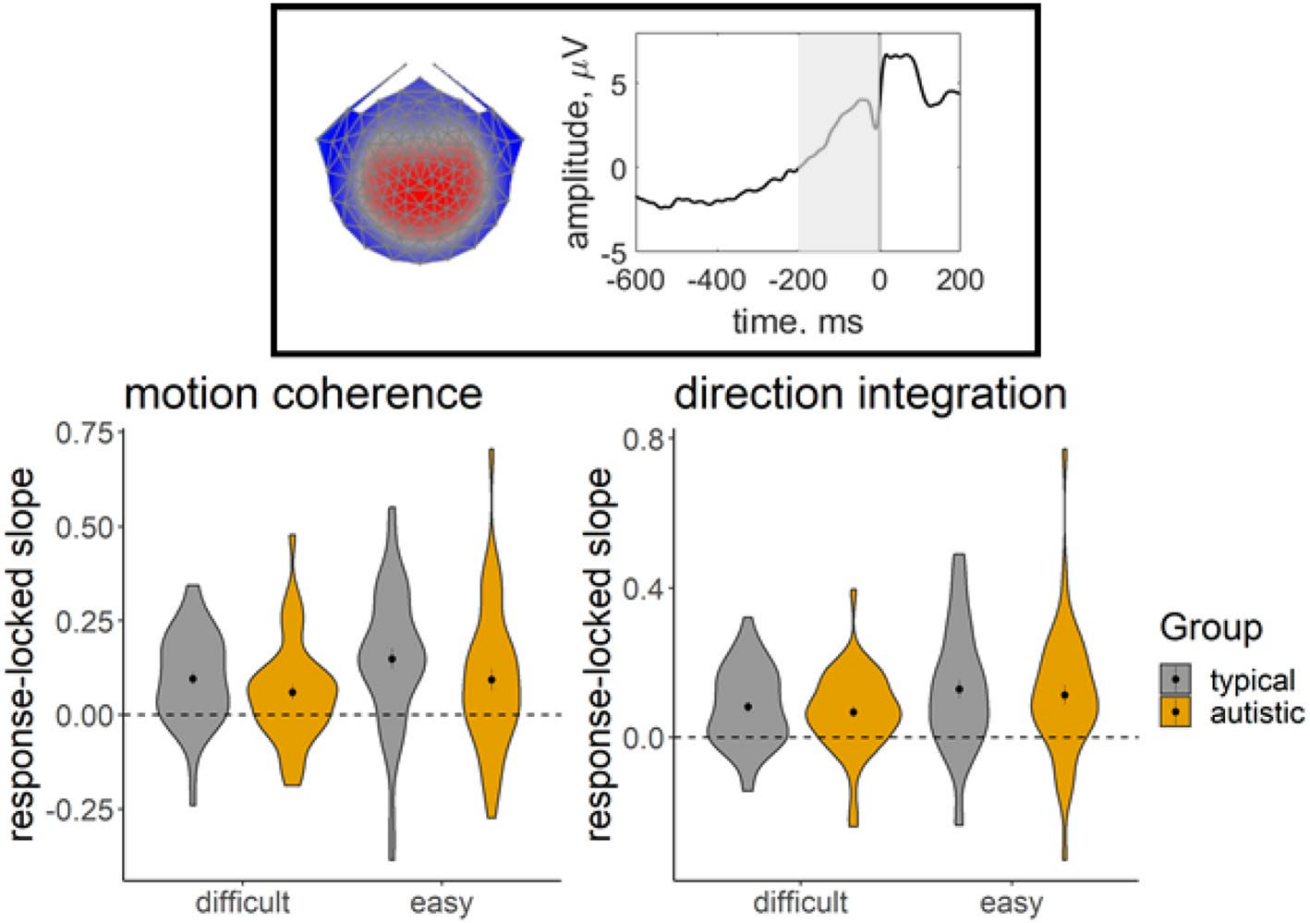
EEG slope measure extracted for inclusion in the joint model. Violin plots showing the kernel probability density for the EEG slope measure extracted for inclusion in the joint model for each group (typically developing: grey; autistic: orange) for each difficulty level. The coloured ‘violins’ reflect the distribution of each group’s data points. The extracted measure was the slope of a linear regression line fitted to each participant’s deconvolved (with regularisation) response-locked waveform, from 200 ms prior to the response to the point of the response (i.e., 0 ms; see shaded area of schematic response-locked waveform in inset). The dotted line reflects a flat slope. Dots and vertical lines represent the group mean and ± 1 SEM.

Figure 6 shows that autistic children had a slightly shallower build-up of activity in the centro-parietal component from 200 ms before the response to the point of the response compared to typically developing children. However, Bayesian ANOVAs revealed no evidence for group differences: the best model of the EEG slope data included only a main effect of difficulty level, for both tasks. When averaging across models, there was insufficient evidence in either task for including a main effect of group (motion coherence: BF = 0.73; direction integration: BF = 0.34) or an interaction between group and difficulty level (motion coherence: BF = 0.63; direction integration: BF = 0.27). Following Manning et al.^31,54^, we expected this EEG measure to relate to drift-rate in a joint model. Supplementary Fig. S5 plots individual estimates of drift-rate against the EEG measure entered into the joint model.

**Figure 7:**
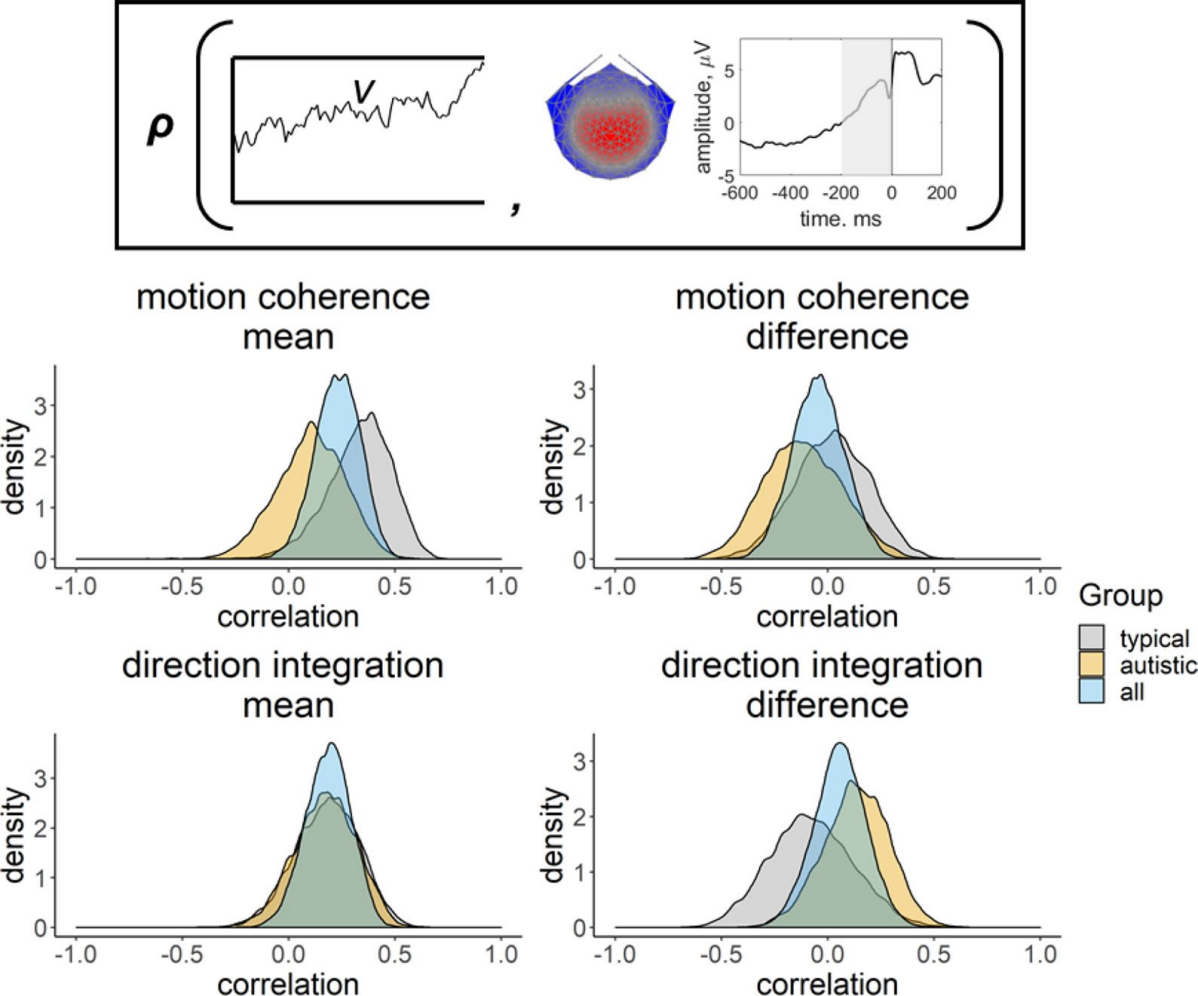
Posterior density plots showing the correlation between drift-rate and the EEG measure. Inset provides a schematic representation of the drift-rate parameter (*v*; left) and EEG measure (slope of response-locked waveform from − 200 to 0 ms around the response; right) that were correlated in the joint model, where ***ρ*** represents the correlation. Posterior density plots in the left column reflect the correlation between the mean drift-rate across difficulty levels *(v.mean)* and the mean EEG slope measure across difficulty levels (*EEG.mean*). Posterior density plots in the right column reflect the correlation between the difference in drift-rate between difficulty levels (*v.diff*) and the difference in EEG slope measure between difficulty levels (*EEG.diff*). Plots for the motion coherence task are presented in the upper row and plots for the direction integration task are presented in the lower row. The blue distribution shows the correlation across all participants, and the grey and orange distributions show separate correlations estimated for typically developing children and autistic children, respectively.

We first fit a joint model where a single correlation between EEG and drift-rate was estimated across all participants, for each task (Fig. 7, blue distribution). There was weak, anecdotal evidence for a positive correlation between the mean EEG measure across difficulty levels and the mean drift-rate across difficulty levels in the motion coherence task (posterior mean correlation = 0.23, 95% credible intervals = [0.02, 0.42], BF = 1.31). The correlation was weaker in the direction integration task, with credible intervals encompassing 0 and BF < 1 (posterior mean correlation = 0.18, 95% CI [− 0.03, 0.38], BF = 0.56). The difference in drift-rate between difficulty levels did not clearly relate to the difference in EEG measure between difficulty levels, with moderate evidence for the null hypothesis (motion coherence task: posterior mean correlation = − 0.04, 95% CI [− 0.28, 0.20], BF = 0.17; direction integration task: posterior mean correlation = 0.06, 95% CI [− 0.19, 0.30], BF = 0.17).

Next we ran joint models where a separate correlation coefficient between EEG and drift-rate was estimated for each group (Fig. 7, grey and orange distributions), in order to see if the relationship could be found in each group (rather than explicitly testing for differences in correlations between groups). In the motion coherence task, there was weak, anecdotal evidence for a positive relationship between mean drift-rate and the mean EEG measure across difficulty levels for the typically developing children (posterior mean correlation = 0.33, 95% CI [− 0.01, 0.59], BF = 1.64), but there was no clear correlation for the autistic children (posterior mean correlation = 0.10, 95% CI [− 0.22, 0.40]) with moderate support (BF = 0.28) for the null hypothesis of no relationship. Moreover, there was moderate evidence for no correlation between difference in drift-rate and difference in EEG measure between difficulty levels in the motion coherence task, for both groups (typically developing: posterior mean correlation = 0.02, 95% CI [− 0.33, 0.35], BF = 0.23; autistic: posterior mean correlation = − 0.12, 95% CI [− 0.46, 0.25], BF = 0.31).

In the direction integration task, there was no evidence for a correlation between mean drift-rate and mean EEG measure across difficulty levels in either group, with anecdotal evidence for the null hypothesis (typically developing: posterior mean correlation = 0.18, 95% CI [− 0.13, 0.46], BF = 0.41; autistic: posterior mean correlation = 0.17, 95% CI [− 0.12, 0.44], BF = 0.35). There was moderate evidence for no correlation between difference in drift-rate and difference in EEG measure between difficulty levels, in both groups (typically developing: posterior mean correlation = − 0.08, 95% CI [− 0.44, 0.31], BF = 0.28; autistic: posterior mean correlation = 0.13, 95% CI [− 0.18, 0.42], BF = 0.30).

### Joint model: ADHD

Finally, we investigated whether diffusion model parameters were related to parent-reported ADHD symptoms, in a series of exploratory, joint models estimating a separate correlation for each group, with each model allowing inattentiveness and hyperactivity/impulsivity to relate to a different model parameter of interest (*v.mean, a, ter*). Scatterplots showing the relationship between ADHD dimensions and parameter estimates from the basic model are provided in Supplementary Figs. S6 and S7. The posterior distributions for the correlations arising from the joint models are presented in Supplementary Figs. S8–S10. In the autism group only, we found weak, anecdotal evidence for a negative relationship between mean drift-rate and hyperactivity/impulsivity for both tasks (motion coherence task: posterior mean correlation = − 0.31, 95% CI [− 0.54, − 0.03], BF = 1.82; direction integration task: posterior mean correlation = − 0.27, 95% CI [− 0.51, 0], BF = 1.27; Fig. S8). For all other relationships, there was relatively more evidence in favour of the null hypothesis (BFs < 1, see Table S1).

## Discussion

In this pre-registered study with a blind modelling approach, we presented autistic and typically developing children with two motion processing tasks that have previously been linked to reduced or increased sensitivity in autism^16–18,50^. To our knowledge, for the first time in the context of autism, we used a combination of approaches that are sensitive to different stages of processing: diffusion modelling and EEG. While the autistic children had slightly increased boundary separation (reflecting increased response caution) compared to typically developing children, the evidence for or against group differences was inconclusive. Moreover, we did not find conclusive evidence to support or refute the hypothesised group differences in evidence accumulation (drift-rate) and non-decision time. We identified a response-locked centro-parietal EEG component, but build-up in this EEG measure was not consistently related to drift-rate in the autism group. While we had not pre-registered hypotheses for relationships between ADHD and diffusion-model parameters, we found weak, anecdotal evidence for a relationship between parent-report measures of ADHD and drift-rate in the autism group, with higher levels of hyperactivity/impulsivity being associated with a lower drift-rate, in both tasks.

First we consider why we did not find evidence for the expected group differences in drift-rate in the two tasks. The autistic children were slightly more accurate and slightly faster than the typically developing children in both tasks, and this was reflected in the autistic children having slightly *increased* drift-rates compared to typically developing children (when controlling for performance IQ), although the evidence was inconclusive, with Bayes factors very close to 1. The small difference between groups was in the direction of our hypothesis for the direction integration task, for which enhanced performance has been previously reported in autistic children of a similar age^18^. Yet, the result was not in the hypothesised direction for the motion coherence task, for which reduced sensitivity has been reported in autistic individuals (see Ref.^14^ for meta-analysis). We note that reduced performance in motion coherence tasks is not consistently found in the literature, e.g.,^18,95,96^, but we know of no reports of enhanced sensitivity. While a range of stimulus parameters could contribute to discrepant results, we think the most important reason that we did not find reduced sensitivity here was because we were not assessing performance at threshold, unlike previous studies assessing motion coherence thresholds in autism, e.g.,^16,17,50^. In the current study, we chose to focus on just two coherence levels, which were selected through piloting to ensure that they would be difficult enough to obtain errors in even the oldest children, as required for diffusion modelling, but easy enough that even the youngest children could do the task. Accordingly, the data were well fit by our diffusion models. Yet, if the main difficulty faced by autistic children during motion coherence tasks is filtering out the randomly moving noise dots^18,24^ and they can even show *enhanced* motion integration abilities when not required to filter randomly moving noise dots^22^, it is conceivable that autistic children may respond similarly, or even with greater sensitivity, relative to typically developing children, at high coherence levels. Future studies could increase the number and range of coherence levels, to investigate whether group differences would emerge at lower levels of coherence.

While group differences in drift-rate are likely to be task dependent, the existing literature has shown relatively consistent differences between autistic and non-autistic participants in boundary separation^41–45^ (but see Ref.^97^, which appears to find little difference in boundary separation between groups for a perceptual task). Our results are in the hypothesised direction, with autistic children having slightly wider boundary separation than typically developing children on average. However, this evidence was inconclusive, with relatively more evidence in favour of the null hypothesis than the alternative hypothesis of group differences in both tasks when controlling for performance IQ. This discrepancy with the literature could reflect differences between tasks, or even task instructions. In our paradigm, we explicitly asked participants to emphasise speed *and* accuracy, and awarded points based on both (i.e., an efficiency score), which differs from other studies that reported group differences in boundary separation. For example, in their study of autistic children, Pirrone et al.^42^ did not appear to instruct participants to emphasise response speed, and no feedback was provided. Therefore, it is conceivable that autistic children have a tendency to emphasise accuracy over speed, but when they are asked to emphasise both speed and accuracy, they are able to modulate their boundary separation to some extent. Future research would be required to further ascertain the effects of instructions on boundary separation in autistic participants, as previously investigated in individuals with ADHD^98^.

We originally hypothesised that autistic children would show increased non-decision time compared to typically developing children, based on Pirrone et al.^41^, but note that more recent research suggests no differences between autistic and non-autistic children in this parameter^42^. In the current study, there was slightly more evidence in favour of the alternative hypothesis of group differences compared to the null hypothesis, with autistic children having slightly *shorter* non-decision times. As non-decision times encompass both sensory encoding and response generation processes, we might expect non-decision time estimates to vary according to the type of stimulus to be encoded (e.g., a single grating in Pirrone et al.^42^ vs. multiple white dots in the current study). Future work will therefore be required to understand whether pre-decision (sensory encoding) and post-decision (response generation) processes differ in autistic and non-autistic participants, for a range of stimuli. Moreover, future research will be needed to determine whether diffusion model parameters relate to the core symptoms of autism.

While we have discussed the evidence for each of our hypotheses separately, we note that many of our Bayes factors were close to 1. As the prior and posterior distributions are similar, the data appear to be relatively uninformative, suggesting that any group differences are small. One factor potentially contributing to the inconclusive evidence obtained across all of our hypotheses, while other studies have reached firmer conclusions, is that we used a conservative method of testing our hypotheses, which takes account of uncertainty in individual participant-level parameters. Instead, other studies, e.g.,^42,44^ conducted inferential statistics on point estimates extracted from the diffusion model fitting procedure, thus not accounting for uncertainty in individual-level parameters, and potentially erroneously inflating the evidence in favour of the winning model^99,100^. In order to overcome the uncertainty associated with individual parameter estimates and provide stronger conclusions, we would need to increase the number of trials collected for each participant (which is challenging when working with children) and/or increase the sample of participants tested (which was not possible within the current study due to resource limitations). While our study does not provide conclusive evidence to support or refute hypotheses regarding decision-making parameters in autistic children, looking ahead, the data from this study could be combined with that from previous studies to inform testing priors for further studies using diffusion models with autistic participants. It might also be informative to reanalyse the data from previous studies using a consistent analytical framework that takes into account the uncertainty of parameter estimates at the individual level, to see what effect this has.

To complement our diffusion modelling approach, we collected high-density EEG during task performance from the majority of participants. We identified a response-locked reliable centroparietal component resembling a component previously related to drift-rate in typically developing children^31^ and children with dyslexia^54^. We used a linear deconvolution method to unmix the overlapping stimulus-locked and response-locked activity and then extracted the steepness of the deconvolved response-locked waveform preceding the response to add to a joint model. Although we have previously found that the slope of this component relates to drift-rate in typically developing children and children with dyslexia in a motion coherence task, here joint modelling showed no evidence of a relationship between the slope of this component and drift-rate estimates in autistic children in the motion coherence task.

While it is possible that this lack of a clear relationship between EEG and drift-rate derives from our choice of EEG analysis method or noise in our EEG recordings, we think this is unlikely given that the previous study of children with dyslexia used the same analytical approach and EEG system and *did* find a relationship^51^. This result is therefore intriguing as it suggests that there may not necessarily be a 1:1 correspondence between the positive centro-parietal component and drift-rate. It has been shown that the centro-parietal positivity also reflects the subjective clarity of perceptual experience^101^, which likely relates to decision confidence. Accordingly, in the monkey brain, parietal neurons involved in decision-making also encode choice confidence^102^, and in humans, the same neural sources in prefrontal cortex and parietal cortex appear to be involved in evidence accumulation and confidence^103^. Atypical confidence judgments have been reported in autistic participants^104–106^, so future research will be required to investigate how confidence relates to our EEG component in autistic and typically developing children.

Another potential reason why there might not be a 1:1 relationship between our EEG component and drift-rate is that, in contrast to the assumption of sequential stages made by the diffusion model, early sensory encoding, evidence accumulation and motor planning may overlap (‘continuous flow’^83,107,108^). If this is the case, the EEG component we identified might reflect more than just evidence accumulation, so that any differences in early sensory encoding and / or motor planning in autistic participants might affect the EEG component while the drift-rate is similar to non-autistic participants: a speculation requiring further investigation. While not the focus of the current investigation, we also note that the early stimulus-locked waveforms (up to ~ 400 ms following stimulus onset) for autistic and typically developing children look very similar (see also Ref.^22^), suggesting that early encoding of evidence (i.e., the input to the decision-making stage) is also not disrupted in autistic children, so future studies could focus on motor planning as an alternative / additional reason why the typical relationship between EEG and drift-rate might not hold in autistic children.

An outstanding issue is the considerable variability in participants’ accuracy and response times, and resulting diffusion model parameter estimates. While the relatively wide age range could be one contributing factor to this variability (see Fig. S11 for parameter estimates plotted as a function of age), we note that substantial variability has also been found previously in a typically developing sample, even within children of the same age group^31^, so there must be other contributing factors too. While some variability in estimates may be attributable to measurement error, the current study suggests at least one area of individual differences relating to variability in task performance: symptoms related to ADHD. In exploratory analyses, the autism group showed weak evidence for a relationship whereby participants with high levels of hyperactivity had lower drift-rates. This result is in line with previous studies showing that individuals with ADHD had lower drift-rates than control participants in a range of tasks^44,64,109–111^. We did not find evidence for a relationship between ADHD symptoms and drift-rate in the typically developing sample, which we suspect is due to a lack of variance within the typically developing group on this measure. Future research could investigate whether this is also the case when using a measure that is designed to better capture variability along a continuum (e.g., Strengths and Weaknesses of ADHD-symptoms and Normal-behavior^112^). In light of our findings, we suggest that future diffusion model studies of autism should consider the moderating effects of co-occurring traits such as ADHD, and investigate which other factors might contribute to variability in parameter estimates.

In the same vein, it is informative to consider the current results in conjunction with a related study of children with dyslexia, which used the same tasks, procedure, statistical analyses and sample size as in the current study^54^. Children with dyslexia showed reduced drift-rates in both tasks: a pattern not seen in autistic children here. This is interesting given that elevated motion coherence thresholds have been reported in both developmental conditions^113^ (see Ref.^15^ for review), with a theory that this reflects an underlying vulnerability in the development of the dorsal stream that is involved in motion processing^15^. By combining the current study and that of Manning et al.^54^, we suggest that motion processing differences are less pronounced in autistic children than they are for children with dyslexia. Complementing this finding, recent meta-analyses suggested a smaller effect size for coherent motion processing differences in autism^14^ than in dyslexia^114^. There seem to be some similarities in how autistic children and children with dyslexia process motion information. For example, both autistic and dyslexic children differ from typically developing children at ~ 430 ms following stimulus onset in an occipital component specifically in the motion coherence task, but not the direction integration task^22^, and both groups of children appear to show slightly reduced amplitudes in the response-locked centroparietal component, as shown in the current study and that of Manning et al.^54^. Yet, the pattern of behavioural performance, diffusion model parameters and relationship between model parameters and EEG differs in the two developmental conditions. Extending this paradigm may help to similarly identify areas of convergence and divergence between other developmental conditions, while also providing a framework to help link brain and behaviour. An additional benefit of the diffusion modelling approach is that sensitivity to sensory information can be compared between developmental conditions while taking account of potential differences in speed-accuracy tradeoffs.

## Supplementary Information

Supplementary Information.

## Data Availability

Analysis scripts and output files are available at: https://osf.io/f3wnt/. Data are available on the UK Data Service: 10.5255/UKDA-SN-855625.
