# Supplementary material for: Behavioural and neural indices of perceptual decision-making in autistic children during visual motion tasks
Source: Sci Rep. 2022 Apr 12;12:6072. doi: 10.1038/s41598-022-09885-4 (PMC9005733; doi:10.1038/s41598-022-09885-4)
Supplement: Supplementary file 1 — Supplementary Information. [file 41598_2022_9885_MOESM1_ESM.pdf]

Supplementary Materials relating to Manning et al. ‘Behavioural and neural indices of perceptual decision-making in autistic children during visual motion tasks’

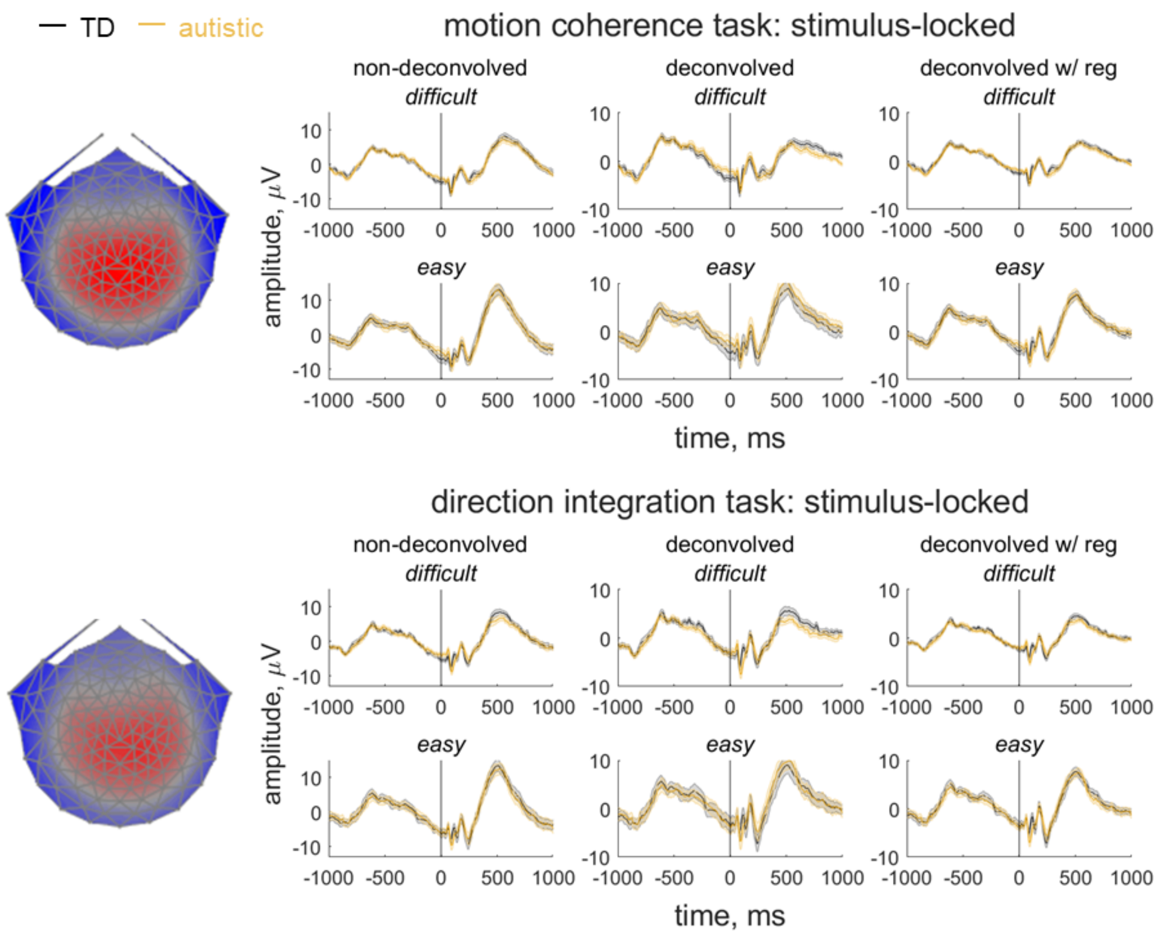

Figure S1. Stimulus-locked evoked potentials for the motion coherence task and direction integration task

Topographic maps represent the forward-model projections of the most reliable component reflecting the weights given to each electrode following reliable components analysis (RCA) on data from all participants pooled across difficulty level for the motion coherence task (top) and direction integration task (bottom). Each individual’s continuous data were multiplied by these weights to provide a component average waveform, with group average stimulus-locked waveforms (±1SEM) shown for typically developing children (TD; grey) and autistic children (orange) for difficult and easy levels. The left column shows non-deconvolved group average waveforms. The central column shows deconvolved group average waveforms (without regularisation). The right column shows deconvolved group average waveforms with regularisation (ridge regression).

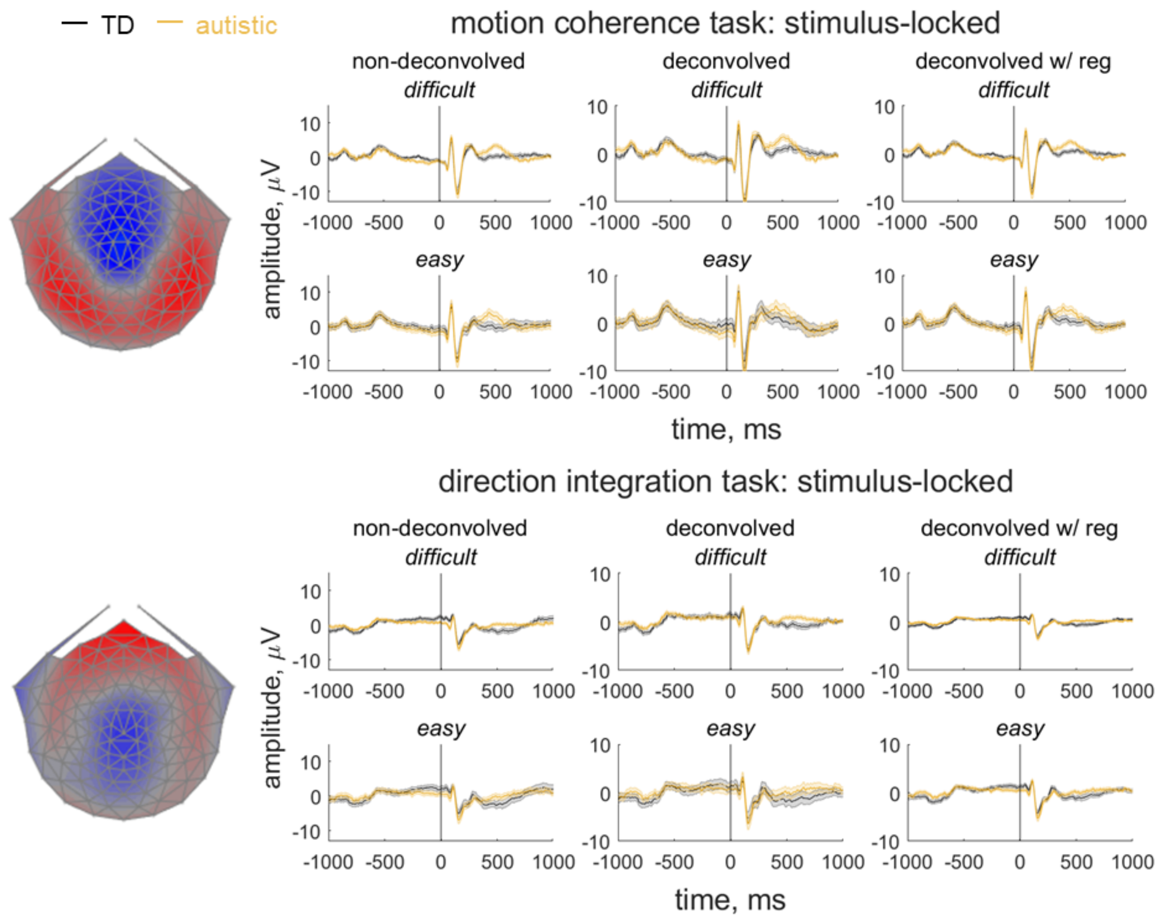

Figure S2. Stimulus-locked evoked potentials for the motion coherence task and direction integration task for the second-most reliable component (RC2)
Topographic maps represent the forward-model projections of the second-most reliable component reflecting the weights given to each electrode following reliable components analysis (RCA) on data from all participants pooled across difficulty level for the motion coherence task (top) and direction integration task (bottom). Each individual's continuous data were multiplied by these weights to provide a component average waveform, with group average stimulus-locked waveforms (±1SEM) shown for typically developing children (TD; grey) and autistic children (orange) for difficult and easy levels. The left column shows non-deconvolved group average waveforms. The central column shows deconvolved group average waveforms (without regularisation). The right column shows deconvolved group average waveforms with regularisation (ridge regression). The autistic children and typically developing children show remarkably overlapping waveforms in early stages of processing (over the first ~400 ms following stimulus onset, see also [35]). Note that the topographic maps and waveform peaks are less defined in the direction integration task than in the motion coherence task.

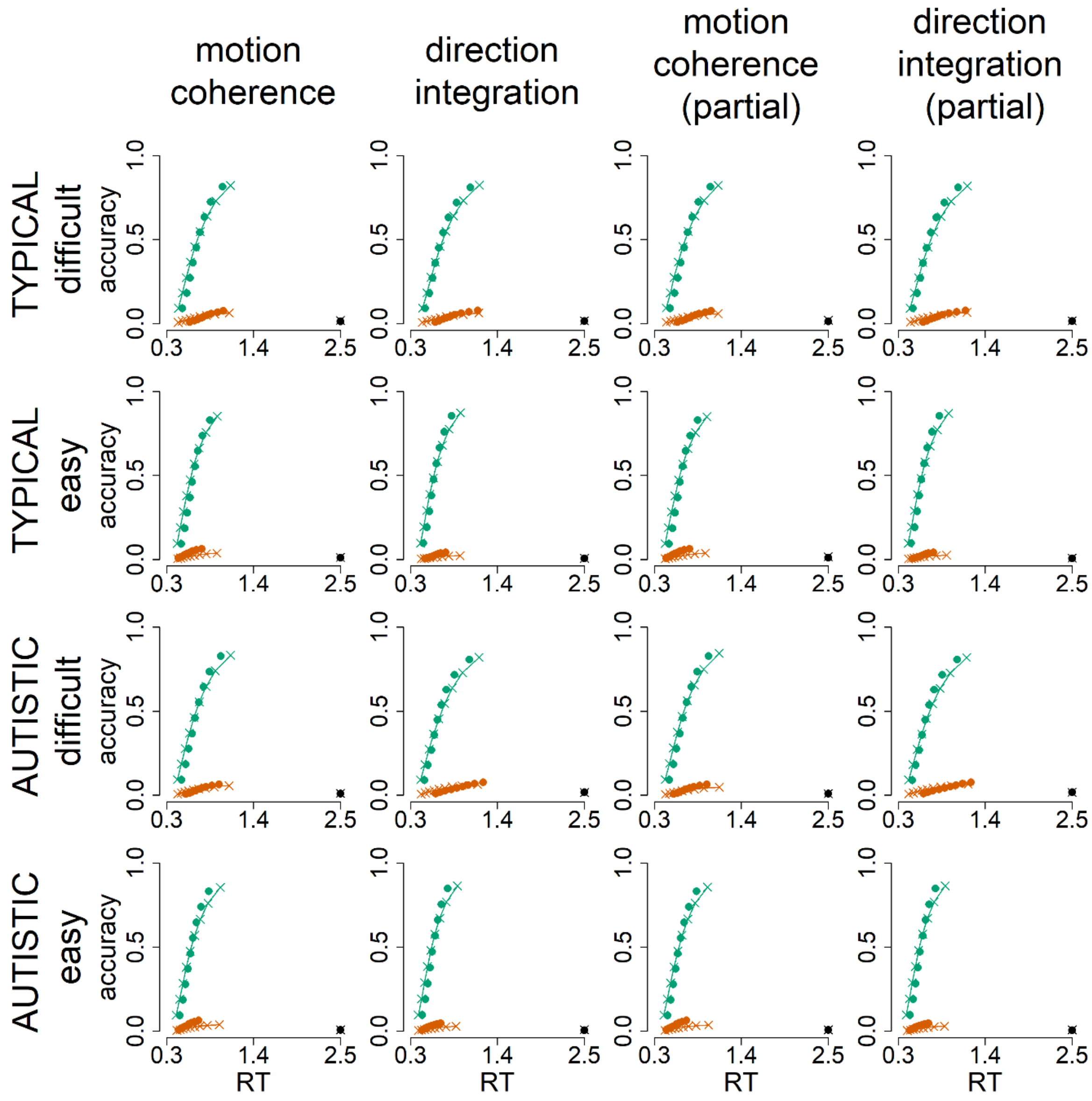

Figure S3. Model fits
Defective cumulative density function plots for each of the four models, for typically developing children (upper rows) and autistic children (bottom rows) for difficult and easy levels. Green represents correct responses and red represents error responses, at each of 9 quantiles. The dots reflect the observed data and crosses with connecting lines reflect the model fit. The dots and crosses at 2.5 seconds reflect the observed and model predicted misses.

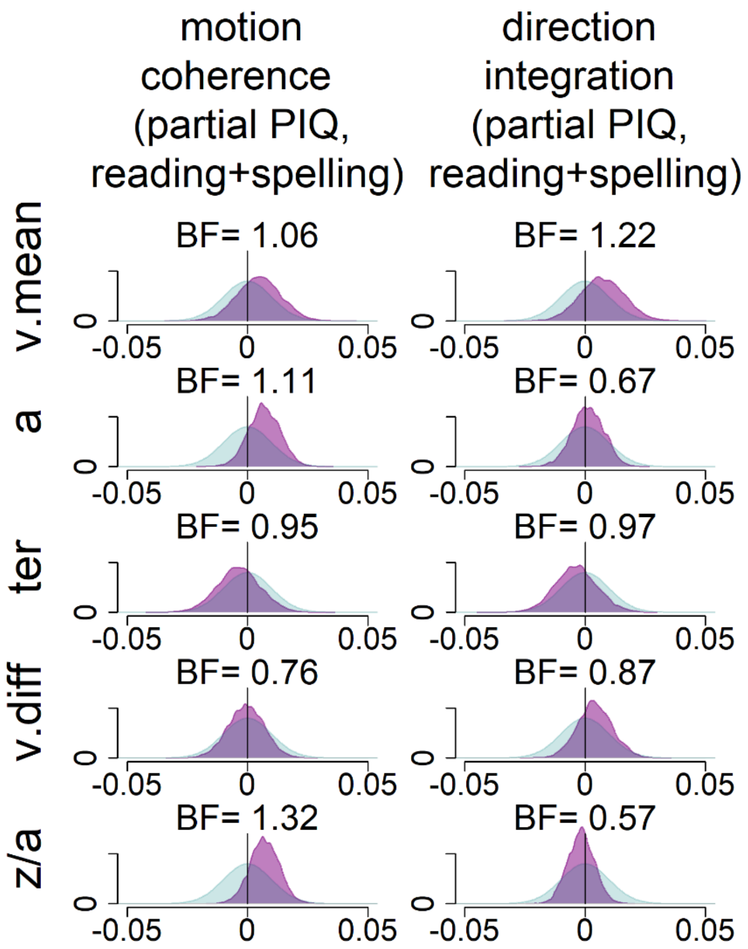

Figure S4. Prior and posterior density distributions for model with reading ability, spelling ability and performance IQ partialled out

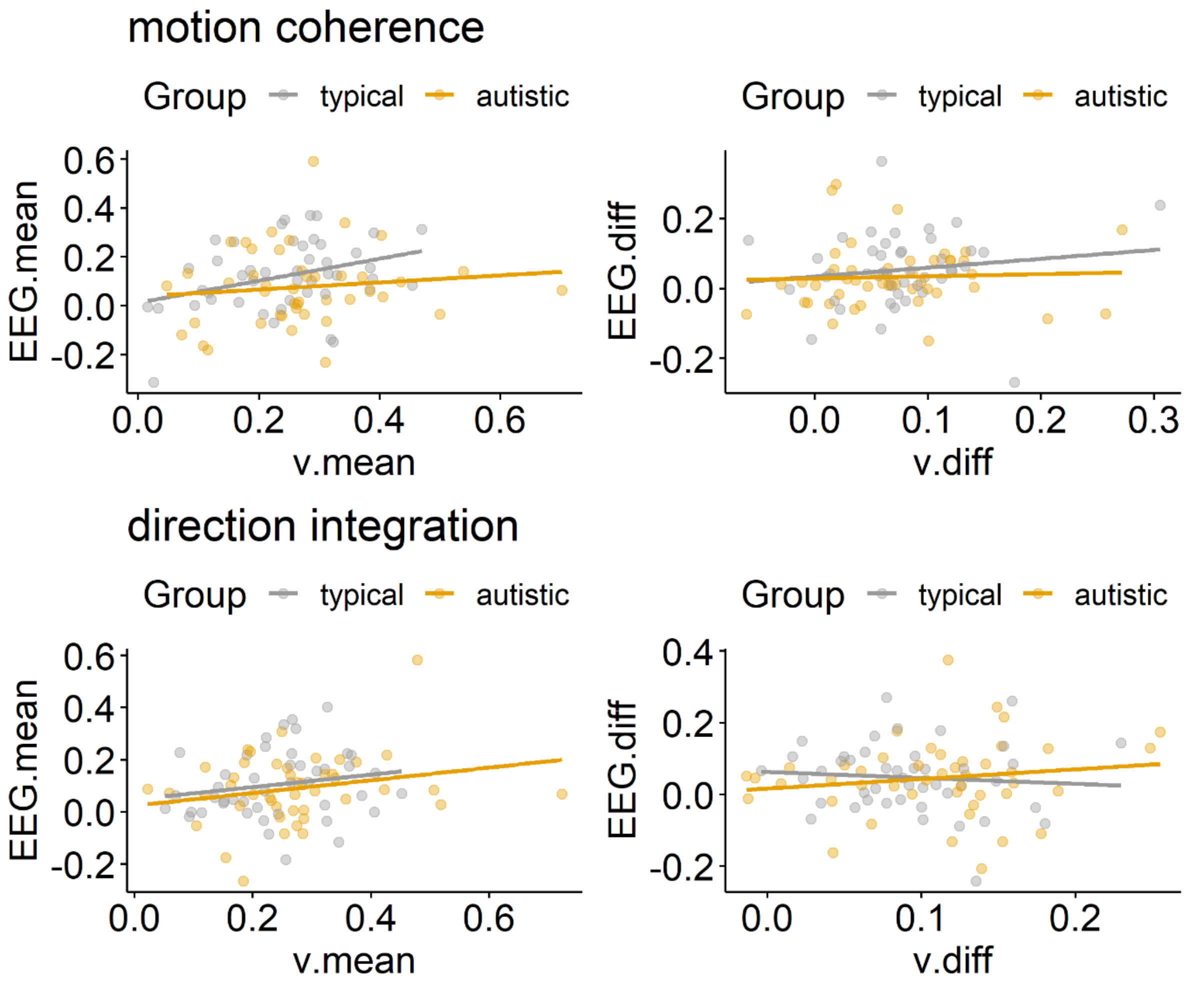

Figure S5. Scatterplots showing relationship between drift-rate and EEG
Left panels show maximum likelihood estimates contained within the posterior for each participant's mean drift-rate across difficulty levels (*v.mean*) plotted against the slope of EEG activity averaged across difficulty levels (*EEG.mean*) for the motion coherence (top) and direction integration (bottom) tasks. Right panels show point estimates for each participant's difference in drift-rate between difficulty levels (*v.diff*) plotted against the difference in slopes of EEG activity between the two difficulty levels (*EEG.diff*), for each task. Typically developing children are plotted in grey and autistic children are plotted in orange.

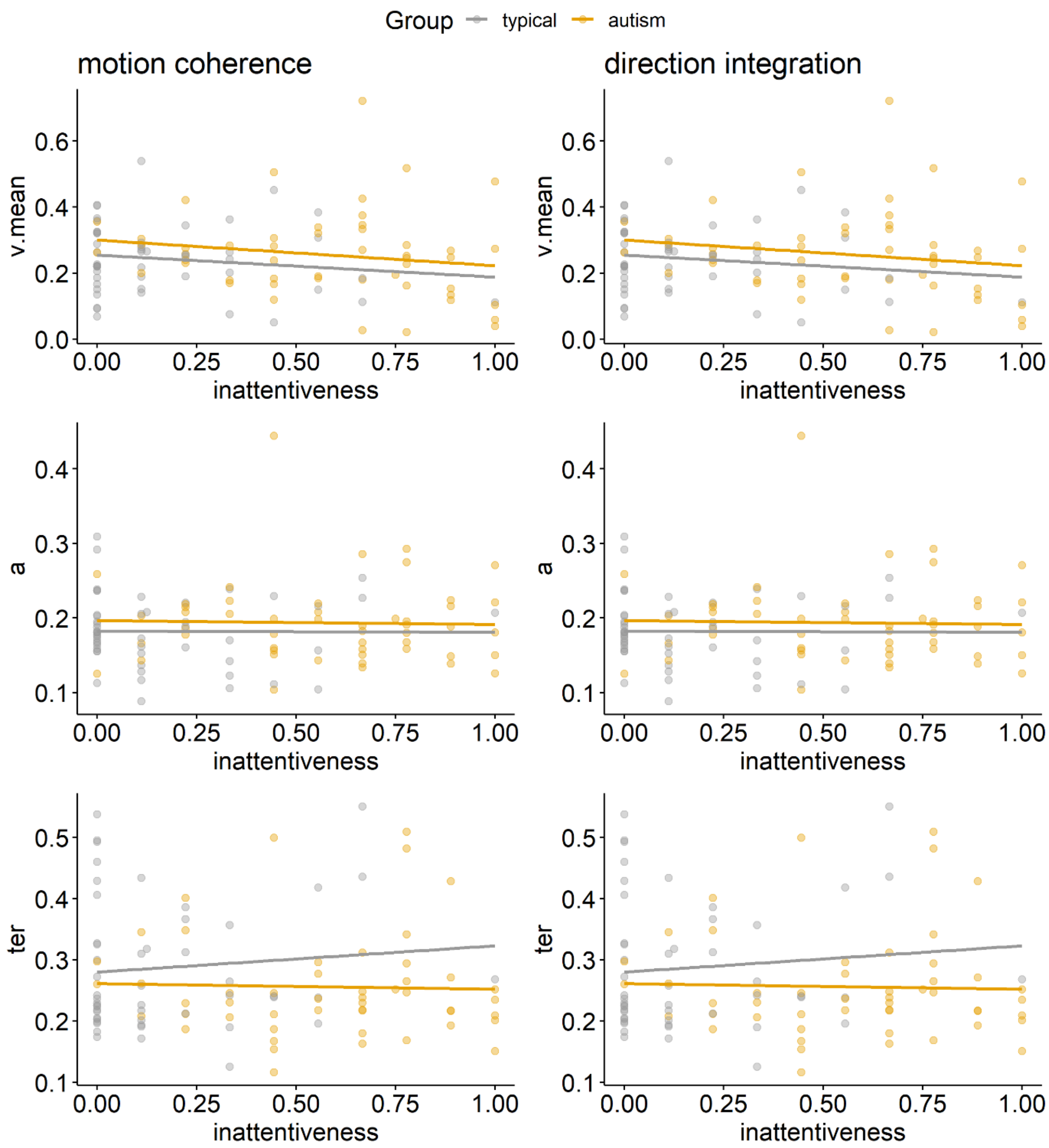

Figure S6. Scatterplots showing relationships between main diffusion model parameters and inattentiveness scores

Maximum likelihood estimates contained within the posterior for each participant's mean drift-rate across difficulty levels (*v.mean*), boundary separation (*a)* and non-decision time (*ter*), as a function of inattentiveness scores, for the motion coherence task (left column) and direction integration task (right column). Typically developing children are plotted in grey and autistic children are plotted in orange.

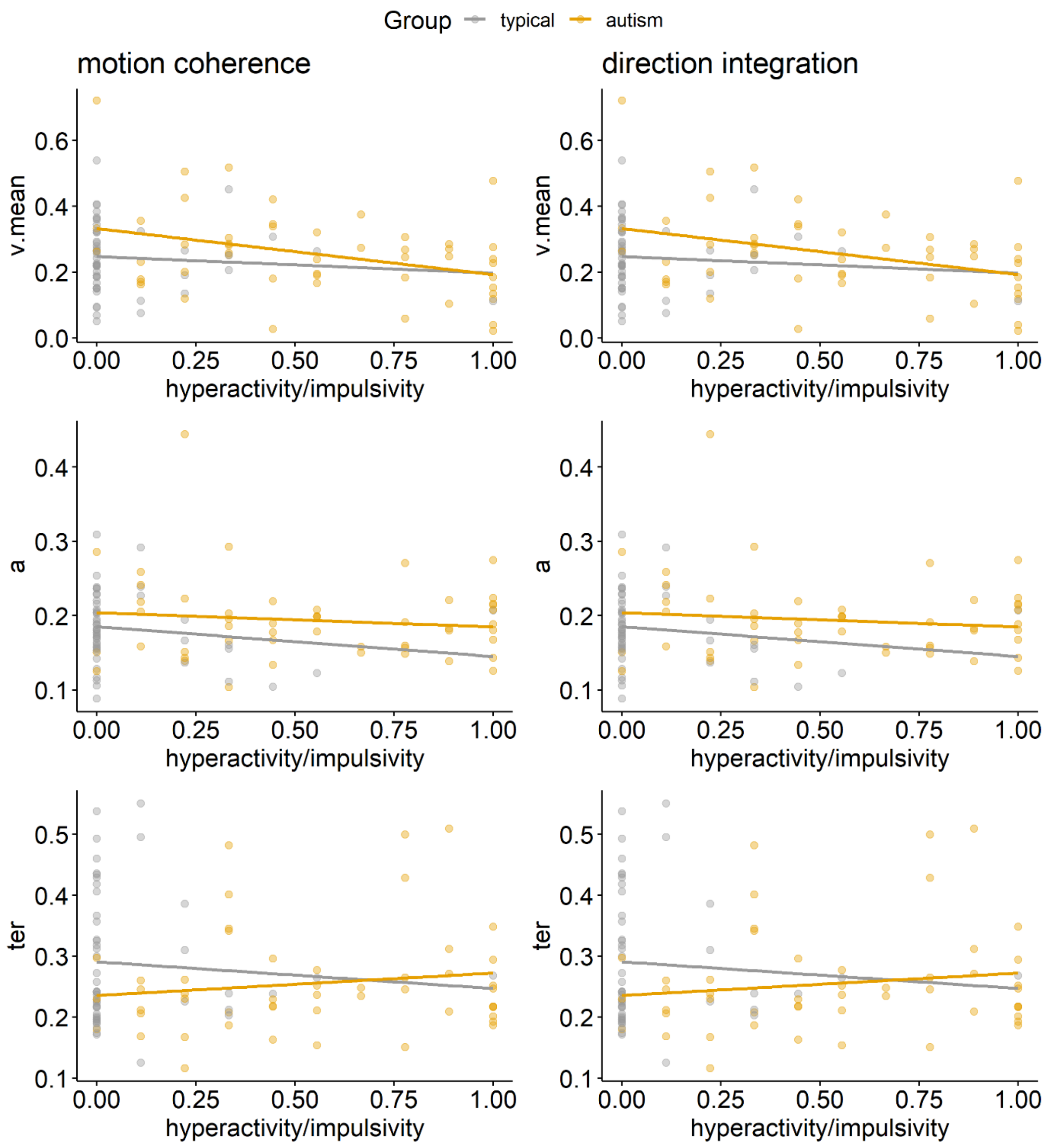

Figure S7. Scatterplots showing relationships between main diffusion model parameters and hyperactivity/impulsivity scores

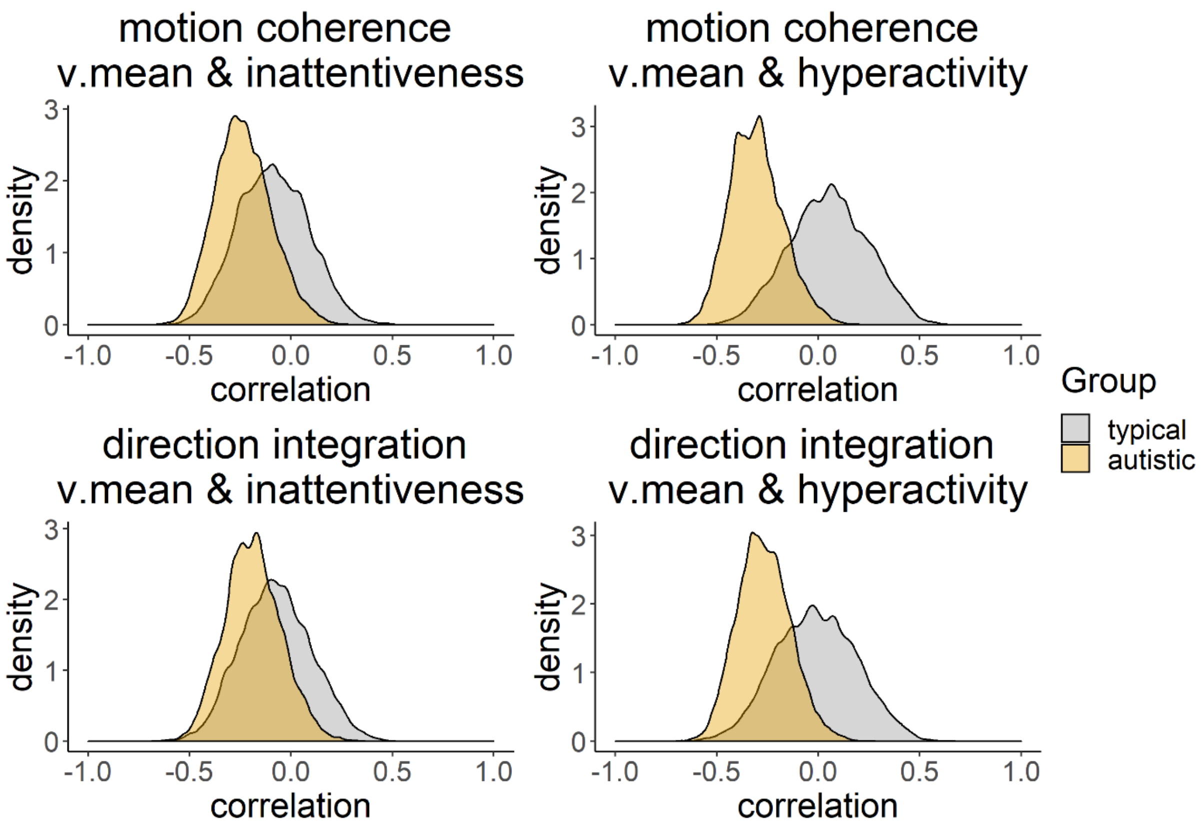

Figure S8. Posterior density plots showing correlations between mean drift-rate across difficulty levels (v.mean) and parent-reported ADHD symptoms

Plots for the motion coherence task are presented in the upper row and plots for the direction integration task are presented in the lower row. The left column shows the relationship between mean drift-rate (v.mean) and inattentiveness, and the right column shows the relationship between mean drift-rate (v.mean) and hyperactivity/impulsivity. The grey and orange distributions show separate correlations estimated for typically developing and autistic children, respectively.

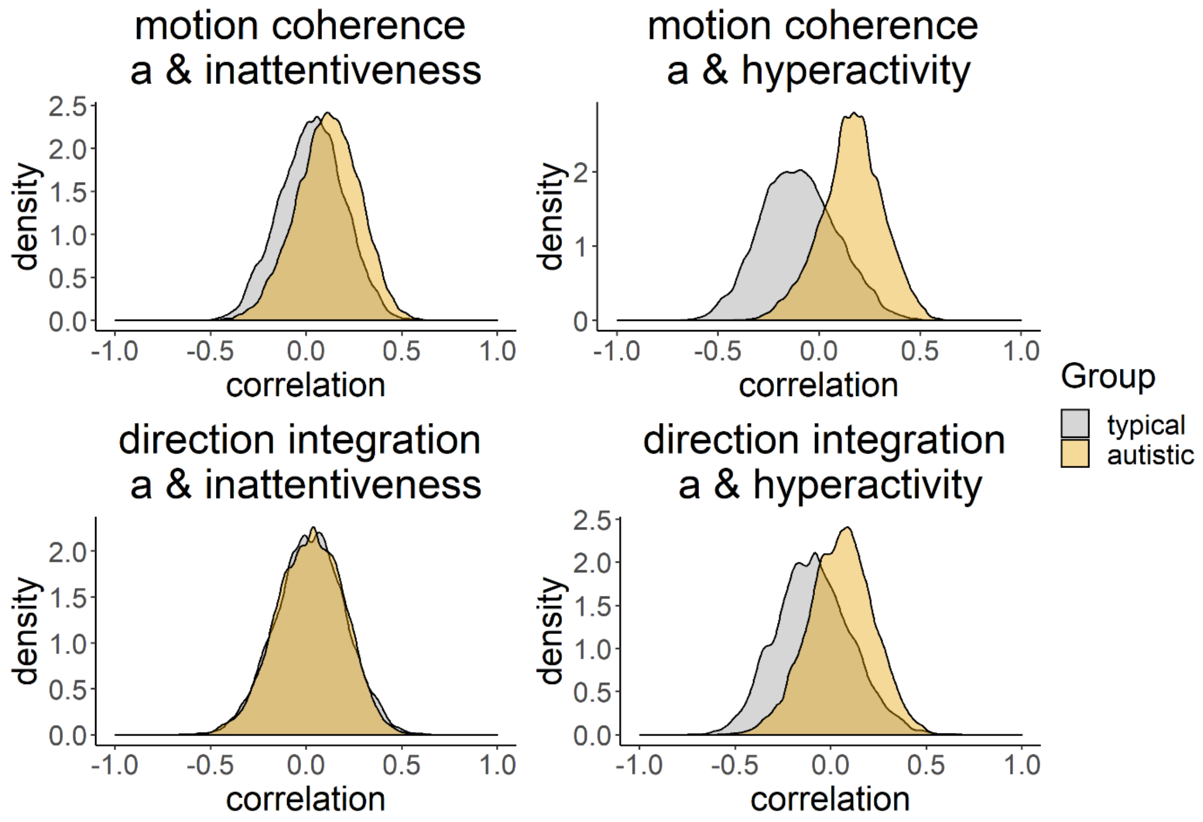

Figure S9. Posterior density plots showing correlations between boundary separation (a) and parent-reported ADHD symptoms

Plots for the motion coherence task are presented in the upper row and plots for the direction integration task are presented in the lower row. The left column shows the relationship between boundary separation (a) and inattentiveness, and the right column shows the relationship between boundary separation (a) and hyperactivity/impulsivity. The grey and orange distributions show separate correlations estimated for typically developing and autistic children, respectively.

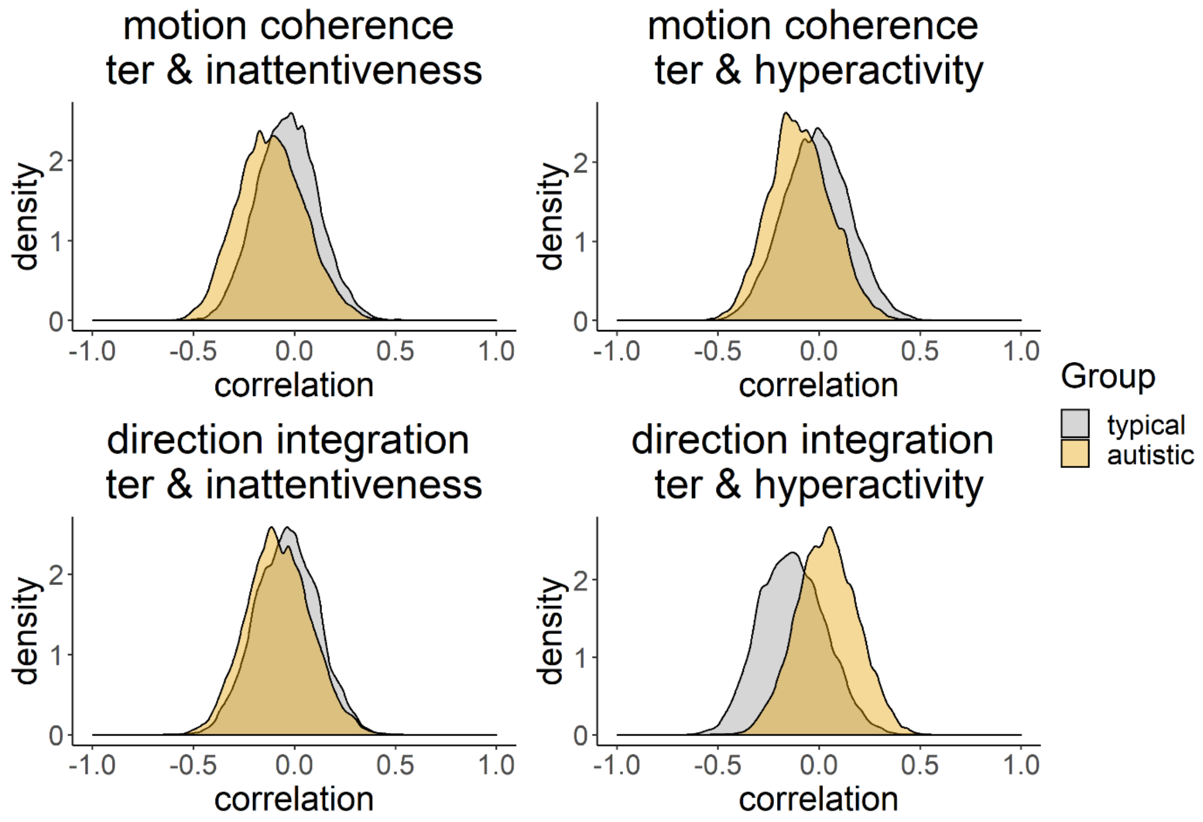

Figure S10. Posterior density plots showing correlations between non-decision time (ter) and parent-reported ADHD symptoms

Plots for the motion coherence task are presented in the upper row and plots for the direction integration task are presented in the lower row. The left column shows the relationship between non-decision time (ter) and inattentiveness, and the right column shows the relationship between non-decision time (ter) and hyperactivity/impulsivity. The grey and orange distributions show separate correlations estimated for typically developing and autistic children, respectively.

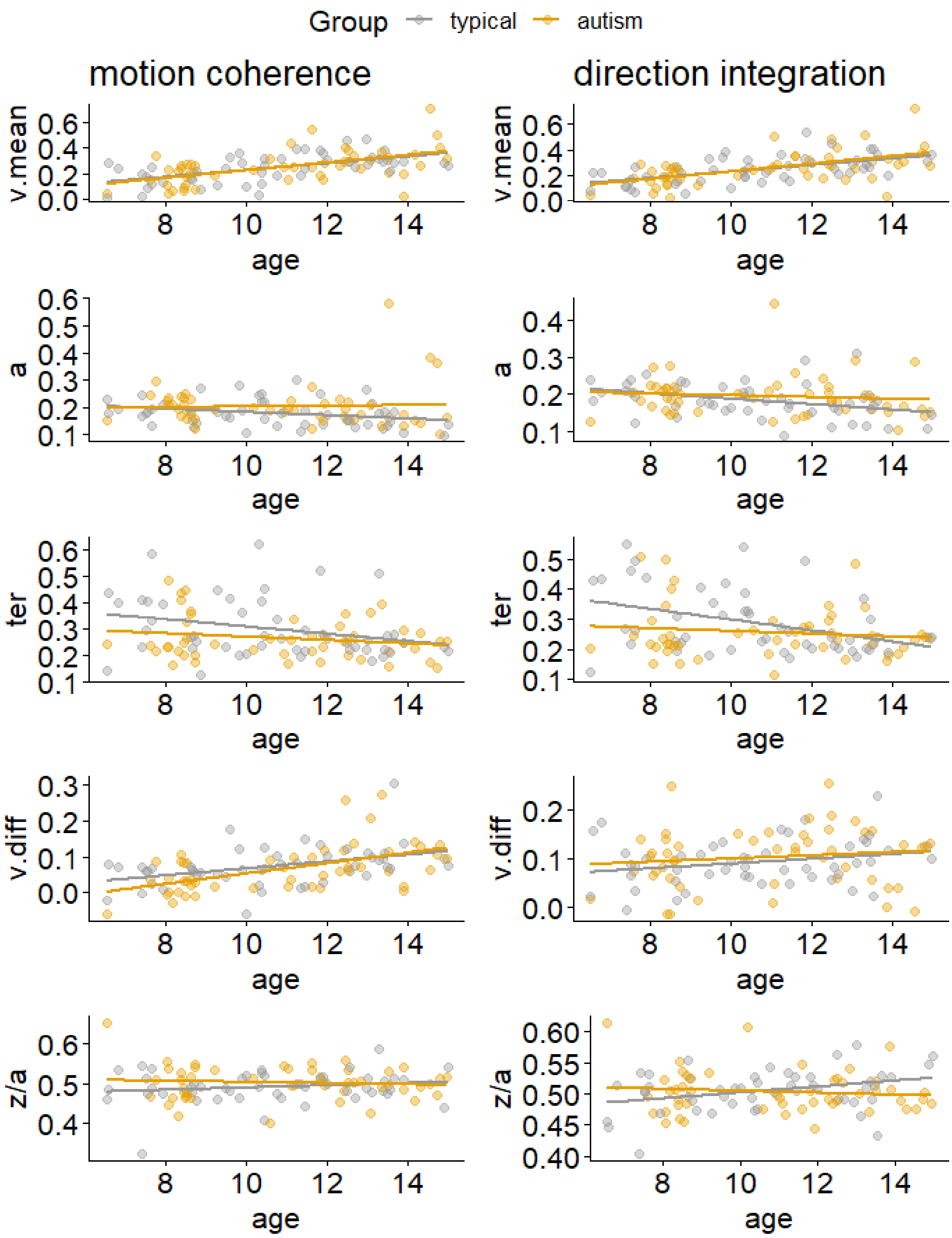

Figure S11. Scatterplots plotting individual parameter estimates against age

Maximum likelihood estimates contained within the posterior for each participant's mean drift-rate across difficulty levels (*v.mean*), boundary separation (*a),* non-decision time (*ter*), difference in drift-rate between difficulty levels (*v.diff*), and starting point *(z/a),* plotted as a function of age, for the motion coherence task (left column) and direction integration task (right column). Typically developing children are plotted in grey and autistic children are plotted in orange.

Table S1. Statistics summarising posterior distributions for the correlations between parent-reported ADHD symptoms (hyperactivity/impulsivity and inattentiveness) and diffusion model parameters, for each group, in each task.

Corr	Motion coherence task		Direction integration task	
	Typical	Autistic	Typical	Autistic
Hyperactivity/impulsivity,				
v.mean	.05 [-.32, .42], .27	-.31 [-.54, -.02], 1.82	-.01 [-.39, .37], .27	-.27 [-.51, 0], 1.27
a	-.10 [-.46, .28], .30	.16 [-.16, .45], .38	-.09 [-.45, .31], .29	.05 [-.29, .37], .24
ter	-.01 [-.33, .31], .21	-.09 [-.38, .22], .25	-.13 [-.43, .19], .30	.04 [-.26, .33], .21
Inattentiveness,				
v.mean	-.09 [-.41, .25], .27	-.23 [-.48, .06], .68	-.07 [-.40, .27], .25	-.19 [-.45, .10], .47
a	.03 [-.30, .35], .22	.11 [-.22, .42], .29	.02 [-.33, .37], .23	.02 [-.33, .35], .24
ter	-.04 [-.32, .27], .20	-.12 [-.42, .22], .28	-.04 [-.34, .26], .20	-.08 [-.38, .24], .23

Note. Data are presented as posterior mean correlation [95% credible intervals], Bayes factor (BF), for the correlation between each diffusion model parameter and ADHD measure.
